# mHealth-Enabled Stroke Screening for Pediatric Sickle Cell Disease in Low-Resource Settings: Systematic Literature Review of Critical Barriers, Emerging Technologies, and AI-Driven Solutions

**DOI:** 10.2196/76937

**Published:** 2026-04-06

**Authors:** Nursat Jahan, Seung Yup Lee, Nafisa Anjum, Monica Swahn, Sangsun Choi, Andrew Peachey, Sweta Sneha, Chitalu Kabwe, Nazmus Sakib

**Affiliations:** 1 College of Computing and Software Engineering, Kennesaw State University, Marietta Campus, J3218 Atrium Building, Marietta, GA, 30060, United States, 1-470-578-3803; 2 Department of Electrical and Computer Engineering, Kennesaw State University, Marietta Campus, Marietta, GA, United States; 3 School of Public Health, Virginia Commonwealth University, Richmond, VA, United States; 4 School of Communication & Media, Kennesaw State University, Kennesaw Campus, Kennesaw, GA, United States; 5 Department of Health Promotion and Physical Education, Kennesaw State University, Kennesaw Campus, Kennesaw, GA, United States; 6 Wright School of Business, Dalton State College, Dalton, GA, United States; 7 Michael A. Leven School of Management, Kennesaw State University, Kennesaw Campus, Kennesaw, GA, United States

**Keywords:** stroke screening, stroke prevention, low-resource setting, pediatric stroke, sickle cell disease, Preferred Reporting Items for Systematic Reviews and Meta-Analyses, PRISMA, mobile health, mHealth

## Abstract

**Background:**

Sickle cell disease (SCD) is a genetic blood disorder affecting millions globally, with life-threatening complications, and most patients live in sub-Saharan Africa. Particularly, children with SCD have a high risk of stroke. Although early screening for stroke could help prevent many cases, access to effective stroke screening remains limited in low-resource settings (LRS). Existing traditional approaches are highly operator-dependent, costly, resource-intensive, or difficult to deploy at scale in pediatric care. These limitations highlight the urgent need for accessible, scalable, and child-appropriate stroke screening and assessment tools suitable for low-resource health care contexts.

**Objective:**

The aims of this systematic literature review are to (1) uncover system-level barriers affecting stroke screening accessibility for patients with pediatric sickle cell disease (PSCD) in LRS, including underserved contexts within high-income countries; (2) identify existing and emerging stroke screening and assessment technologies and their implementation characteristics, such as feasibility, scalability, portability, and training requirements; and (3) propose a user-centered mobile health (mHealth) framework for stroke screening that improves accessibility and feasibility in resource-constrained health care settings.

**Methods:**

PRISMA (Preferred Reporting Items for Systematic Reviews and Meta-Analyses) guidelines were followed to organize the search process. A systematic search was conducted using an advanced query and defined eligibility criteria in the academic databases of PubMed, IEEE Xplore, Wiley Online Library, and Google Scholar. Studies published in English between January 1, 2021, and October 31, 2025, were selected. Collected data were arranged in a preformatted Microsoft Excel spreadsheet for analysis. Risk-of-bias assessment was performed using various risk-of-bias assessment tools because of the heterogeneity of the included studies. Narrative synthesis was used for data synthesis.

**Results:**

The literature search initially identified 1465 studies, of which 28 (2%) were selected for analysis. Among the 28 studies, 10 (36%) focused on stroke screening accessibility for patients with PSCD in either low- and middle-income countries or other income-level countries for LRS, and 18 (64%) outlined key features and the feasibility of stroke screening technologies. Identified barriers were organized into 4 major categories (workforce and training constraints, health care system and infrastructure barriers, sociocultural and awareness factors, and economic and logistical constraints), emphasizing difficulties in accessing stroke screening in LRS. Additionally, existing and emerging stroke screening technologies were classified into 5 groups: nonimaging, imaging, light-based optical spectroscopy, biomarker-based, and artificial intelligence– and machine learning–based mHealth wearable approaches. Finally, a comprehensive mHealth app is proposed for an easy-to-use screening experience to address stroke screening challenges for patients with PSCD in LRS.

**Conclusions:**

This study contributes to identifying major barriers to stroke screening in LRS and highlights key characteristics of stroke screening solutions that can be used in the future. It also contributes to the design of a holistic mHealth solution for implementing stroke screening clinical care for patients with PSCD in LRS.

## Introduction

### Background

Sickle cell disease (SCD) is a genetic blood disorder affecting millions of people globally, most of whom live in sub-Saharan Africa. About 75% of patients with SCD are from this region and experience high mortality rates, with an estimated 50% to 90% of affected children dying before the age of 5 years due to infections or life-threatening complications [1,2]. Children with SCD often experience severe complications. Studies indicate that about 11% of children with SCD in these regions experience stroke by the age of 20 years [3,4]. For instance, each year, at least 20,000 babies are born with SCD in Uganda, and up to 80% of these children do not survive beyond the age of 5 years because of complications and limited access to adequate health care [4,5]. Weak health care infrastructure, high costs, and limited availability of specialized medical personnel in these low-resource settings (LRS) further compound the challenges faced by these children. Particularly in rural regions, health care disparities present significant obstacles [5-7]. Despite ongoing efforts, mortality among children with SCD remains high because of inadequate and delayed stroke screening, emphasizing the urgent need for affordable and accessible solutions in LRS [5,8].

Several stroke screening tools exist for pediatric patients with SCD, differing in mechanism, accessibility, and clinical requirements. These stroke screening and assessment technologies include nonimaging techniques such as transcranial Doppler (TCD), the current gold standard; imaging methods such as magnetic resonance imaging (MRI) or magnetic resonance angiography (MRA); biomarker-based approaches; light-based optical spectroscopy techniques such as speckle contrast optical spectroscopy (SCOS), an emerging noninvasive technology; and artificial intelligence (AI)- and mobile health (mHealth)-enabled solutions for early stroke detection.

Traditional approaches, such as TCD ultrasonography, are the gold standard for stroke screening in pediatric sickle cell disease (PSCD) and can assess stroke risk in children with SCD aged 2 to 16 years [9]. Few centers in sub-Saharan Africa have the neuroradiology facilities needed to conduct TCD screenings. A clinical trial in Nigeria revealed that only around 3000 TCD assessments were performed out of 40,000 children needing screening, covering less than 8% [10]. Regular TCD detection of abnormal blood flow in the brain has proven effective in reducing stroke incidence in these children [11]. Studies have shown that consistent TCD screening can drastically lower stroke rates [3,12]. However, TCD has 2 major drawbacks: its operation is highly dependent on the expertise of operators, and although it is effective for assessing major brain arteries, it is not suitable for examining blood flow in smaller or specific parts of the brain [13]. For these reasons, despite its efficacy, TCD is still not widely implemented in resource-limited settings.

MRI or MRA is another crucial tool for stroke screening, delivering detailed images that reveal silent cerebral infarcts (SCI) and other cerebrovascular problems [14]. Research shows that MRI provides a broader view of cerebrovascular changes than TCD, leading to earlier interventions and better outcomes [15]. However, MRI is often unavailable in low-resource areas because of its high cost and lengthy availability procedures [16]. Moreover, the need for dye contrast and sedation in children adds complexity to its use. Hence, more affordable and less cumbersome imaging techniques are necessary as alternatives to MRI [14].

Recent advances in biomarker and genomic research now enable stroke risk assessment in patients with SCD [17]. Biomarker screening quantifies proteins and inflammatory markers, while genomic screening identifies genetic variations linked to susceptibility [18]. However, research on biomarkers and genomic screening for stroke prevention in SCD is ongoing [19].

Light-based SCOS solutions have shown promise in noninvasively assessing stroke risk [20]. Although this approach demonstrates significant potential for accessible and scalable stroke risk assessment, SCOS remains an emerging technology that requires further clinical validation for real-world use [21].

In addition, emerging technologies, such as wearable devices, have great potential for managing SCD because they allow continuous monitoring of vital parameters, such as oxygen saturation and heart rate [22,23]. These devices can alert both patients and health care professionals, enabling timely interventions before complications become life-threatening [24]. Although wearable devices vary in affordability, their continuous use may be impractical in resource-limited settings because of factors such as discomfort from wearing them all the time, the need for regular charging, and unreliable access to electricity [25].

mHealth technologies can significantly impact the management of SCD, especially in LRS [26]. For instance, mobile apps can monitor symptoms such as pain episodes [27,28], track disease progression, and enable timely interventions while also supporting patient engagement [29-31]. Overall, mHealth solutions have great potential in preventing chronic diseases because they can continuously track vital signs such as oxygen saturation and heart rate, providing real-time health information for improved disease management in critical situations [32].

Nevertheless, despite the great potential of mHealth solutions, significant gaps remain, particularly regarding their application to SCD [33,34]. Additionally, it is imperative to identify the barriers that impede the adoption of these mHealth solutions in LRS [35]. Furthermore, these mobile apps could foster better patient engagement in care by encouraging patients to actively participate in managing their condition [36-38].

### Objective

**Figure 1.**
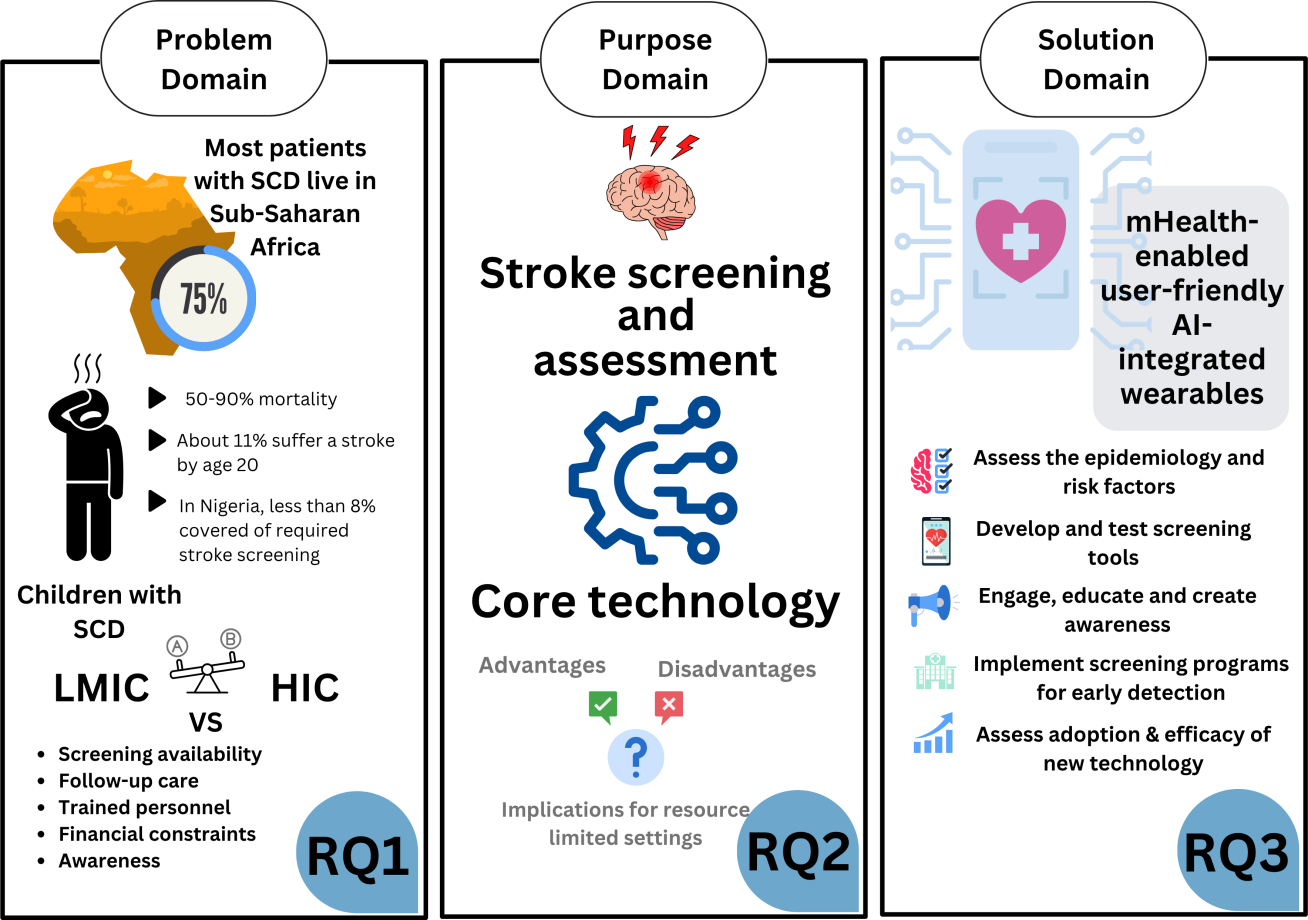
Objective of this study and research question (RQ) generation, organized around the problem domain, purpose domain, and solution domain. AI: artificial intelligence; HIC: high-income country; LMIC: low- and middle-income country; mHealth: mobile health; SCD: sickle cell disease.

Therefore, this study aims to explore the key barriers to stroke screening implementation for pediatric patients with SCD in LRS. We examine the feasibility of conventional and emerging practices for stroke screening assessment and investigate how a more modular, AI-enabled mHealth solution can promote early detection and management, support patient involvement, enhance monitoring, and improve overall health outcomes for pediatric patients with SCD. The findings of this systematic literature review are centered around the following 3 research questions, as illustrated in Figure 1.

The study addressed the following research questions (RQs) as follows:

1. Problem domain (PSCD stroke): What are the key barriers influencing the implementation of stroke screening for patients with PSCD in LRS, including both low- and middle-income countries (LMICs) and resource-constrained contexts within other income-level countries?
2. Purpose domain (stroke screening and assessment): What existing and emerging stroke screening and assessment technologies have been evaluated in individuals with PSCD or broader populations with stroke, and what implementation characteristics (feasibility, scalability, portability, and training requirements) are relevant to their potential use in PSCD stroke screening?
3. Solution domain (addressing aims through mHealth solutions): How can an integrated, user-centered mHealth-based stroke screening framework be proposed to address accessibility barriers and support early stroke detection and follow-up care for patients with PSCD in LRS?

## Methods

### Overview

This systematic review was conducted according to the PRISMA (Preferred Reporting Items for Systematic Reviews and Meta-Analyses) guidelines (Checklist 1). This review was registered with PROSPERO (CRD420251172487) and the full protocol can be accessed through the PROSPERO database. No amendments to the protocol were made following registration.

### Eligibility Criteria

Studies were eligible for inclusion if they met the population, intervention, comparison, and outcome (PICO) criteria outlined in Table 1. Table 1 summarizes the inclusion and exclusion criteria based on the PICO framework, as described in Textbox 1.

**Table 1.** Summary of inclusion and exclusion criteria.

Criteria	Inclusion criteria	Exclusion criteria
Population	Pediatric patients (<18 y) at risk of stroke and diagnosed with SCD^a^. Studies not exclusively focused on PSCD^b^ were included only if they provided indirect, contextual, or technology-enabling evidence relevant to stroke screening implementation in PSCD or low-resource settings.	Studies not focusing on stroke screening among patients with SCD. Studies focusing exclusively on adult populations without relevance to pediatric or implementation contexts.
Intervention	Any stroke screening or assessment method, including: Imaging: MRI^c^ and MRA^d^ Nonimaging: TCD^e^ Laboratory biomarkers Optical spectroscopy or SCOS^f^ tools Wearable devices mHealth^g^ tools AI^h^- and ML^i^-based stroke assessment models Studies not exclusive to PSCD were included only when they provided insights into implementation, scalability, or accessibility, offering possibilities for adaptation in stroke screening of PSCD in low-resource settings.	Studies unrelated to screening interventions (treatment-only studies).
Comparison	No comparator was required because the focus was on narrative and thematic synthesis.	N/A^j^
Outcome	Reports on at least one outcome related to: Screening feasibility or accessibility Accuracy or reliability Workflow or training requirements Implementation barriers or facilitators Portability, cost, or infrastructure needs	Studies not reporting any outcome related to screening implementation or feasibility.
Study design	Quantitative, qualitative, mixed methods, or observational studies. Retrospective cohort studies, cross-sectional studies, implementation studies, narrative reviews, systematic reviews, or technical feasibility studies. Peer-reviewed journal articles or conference papers. Preprints were included only when they provided unique technology-enabling insights not yet available in peer-reviewed literature and were clearly labeled as preprints. Published between January 1, 2021, and October 31, 2025.	Editorials, interviews, comments, unstructured observations, and position papers.

a SCD: sickle cell disease.

b PSCD: pediatric sickle cell disease.

c MRI: magnetic resonance imaging.

d MRA: magnetic resonance angiography.

e TCD: transcranial Doppler.

f SCOS: speckle contrast optical spectroscopy.

g mHealth: mobile health.

h AI: artificial intelligence.

i ML: machine learning.

j N/A: not applicable.

Textbox 1. **Population, intervention, comparison, and outcome (PICO) framework for search query generation**

**Population**

- Direct: pediatric patients (<18 y) who are at risk of stroke, particularly diagnosed with SCD.
- Indirect: Broader populations with stroke or neurological disorders were included only when studies provided indirect, technology-enabling, or contextual evidence relevant to stroke screening implementation in low-resource settings.

**Intervention**

- Studies examining stroke screening or assessment methods, including imaging (magnetic resonance imaging and magnetic resonance angiography), nonimaging approaches (transcranial Doppler), blood biomarkers, laboratory markers, wearable devices, mobile health tools, artificial intelligence– and machine learning–based technologies, or emerging screening modalities

**Comparison**

- No comparator was required because the focus was on narrative and thematic synthesis.

**Outcome**

- Studies reporting on screening feasibility, accessibility, accuracy, workflow processes, implementation challenges, barriers, facilitators, or overall effectiveness of stroke screening methods.

### Definition of LRS

In this review, LRS was defined by the health care delivery context in which the essential capacity for routine stroke screening is hindered by a functional lack of medical infrastructure, specialized workforce, accessible funding for screening, or socioeconomic disparities [39]. This is a setting-level construct and may occur in both LMICs and underserved contexts within high-income countries (HICs) and other income-level countries. Country income status, such as LMIC, HIC, or other income-level countries, was recorded separately as a national-level descriptor.

### Operationalization of Resource Setting

Although national income categories (LMIC or HIC) are frequently used as proxies for resource context, they do not capture the full range of constraints affecting health care delivery. Existing studies conceptualize that “LRS” consists of one or multiple interconnected domains of constraints, such as financial pressure, suboptimal health care service delivery, underdeveloped infrastructure, paucity of knowledge, research challenges and considerations, restricted social resources, geographical and environmental factors, human resource limitations, and the influence of beliefs and practices [39]. Using this concept, studies were classified as conducted in LRS if one or more system-level challenges hindered the routine delivery of stroke screening during the full-text screening and data extraction process. Although studies were initially tagged as conducted in LMICs or HICs using World Bank income classifications, the final classification was guided by this system-level conceptual framework rather than country income alone.

### Database Selection and Search Strategy

The search was conducted in electronic databases including PubMed, Wiley Online Library, IEEE Xplore, and Google Scholar. A predefined search strategy, incorporating both Medical Subject Headings (MeSH) terms and free-text keywords, was used. The search terms were developed to capture literature related to stroke screening barriers, particularly those related to PSCD, stroke screening technologies, mHealth apps, and stroke screening challenges in LRS. Studies published in English between January 1, 2021, and October 31, 2025, were included. An advanced search string was built to search the databases. Search strategies were employed to align with the research questions, ensuring comprehensive coverage of the relevant literature.

The advanced search string used in this search was ((“stroke screening” OR “stroke risk assessment” OR “stroke prevention”) AND (“pediatric” OR “children” OR “child” OR “adolescent”) AND (“sickle cell disease” OR “SCD” OR “sickle cell anemia”) AND (“barriers” OR “enablers” OR “implementation” OR “training”)) OR ((“stroke screening” OR “stroke risk assessment” OR “stroke prevention”) AND (“TCD” OR “Transcranial Doppler” OR “mobile health” OR “mHealth” OR “wearable technology” OR “wearables” OR “wearable” OR “artificial intelligence” OR “AI” OR “MRA” OR “MRI” OR “Magnetic Resonance” OR “machine learning” OR “ML” OR “blood biomarkers” OR “biomarkers”)) (With filter in PubMed: Free full text filter, Wiley online library: Open Access Content).

Resource-setting terminology such as “low-resource,” “resource-limited,” “underserved,” or “resource-constrained” was not used as a mandatory search filter because these labels are not standardized and are applied inconsistently across disciplines and regions and are often absent from titles and abstracts. Instead, identification of LRS was prespecified as part of the full-text screening and data extraction process, using predefined system-level criteria described in the “Eligibility Criteria” section. This approach was used to avoid missing relevant studies while ensuring systematic and reproducible classification of the resource context.

### Selection Process

The screening process was conducted according to the PRISMA framework [40]. Two reviewers (NJ and NA) independently conducted the literature screening process. During the identification step, we gathered our search results from the selected databases and exported them into the reference management tool Zotero (Corporation for Digital Scholarship) to remove duplicates [41]. During the screening and eligibility steps, a title-based screening was conducted using keywords derived from the advanced search queries. Following this, the screening was conducted based on an abstract review. Finally, studies meeting the initial criteria were subjected to full-text review based on the inclusion and exclusion criteria. Both reviewers performed these steps. Discrepancies were resolved through discussion or adjudication by a third reviewer (NS).

### Risk-of-Bias Assessment

Risk-of-bias (RoB) assessment was conducted to evaluate the methodological quality of the included studies. Because the review comprised diverse study designs, including qualitative, quantitative, mixed methods, and technology-focused feasibility studies, the appropriate appraisal tools were applied accordingly. We used the Joanna Briggs Institute (JBI) Qualitative, JBI Analytical Cross-Sectional, JBI Text and Opinion, JBI Systematic Review and Research Syntheses, Newcastle-Ottawa Scale Cohort, Quality Assessment of Diagnostic Accuracy Studies–2 (QUADAS-2), Prediction Model Risk of Bias Assessment Tool (PROBAST), RoB 2.0, and Mixed Methods Appraisal Tool (MMAT; McGill University) for RoB assessment purposes (Multimedia Appendix 1). The assessment focused on key domains, including clarity of aims, methodological rigor, sampling, data collection, analytical transparency, and relevance to clinical or technological implementation. Studies were evaluated independently by 2 reviewers (NS and NA). Disagreements between the 2 reviewers were resolved through discussion.

### Data Extraction

Data extraction was performed independently by 2 reviewers using a standardized Microsoft Excel form. The extracted data included study characteristics (author, year, country, study design, sample size, and population type; Multimedia Appendix 2). We also extracted study details and intervention details (resource settings, stroke screening accessibility challenges, barrier categories, tool type, tool characteristics, training requirements, outcomes, and challenges; (MultimediaAppendices 3  4). Extracted data were cross-checked for consistency and completeness. Due to the variation in methodologies across studies, conducting a meta-analysis was considered unsuitable. Instead, information was narratively synthesized. Data extraction was conducted manually from a total of 28 included studies (27 peer-reviewed studies and 1 preprint) following the identification and selection process outlined in the PRISMA framework.

### Data Synthesis

For this systematic literature review, due to the heterogeneity in interventions, outcomes, and contexts, we employed narrative synthesis, as a meta-analysis was not feasible based on the reviewed studies. As this review did not involve statistical hypothesis testing or meta-analysis, no *P* values were reported. No statistical effect measures, such as risk ratios or mean differences, were used because the outcomes were qualitative and heterogeneous. We narratively synthesized qualitative data related to our research questions, applied thematic analysis to categorize different barriers to stroke screening accessibility, and used narrative synthesis to highlight the key characteristics and outcomes of technological solutions for stroke screening. We categorized included studies as direct evidence, which pertains to clinical stroke screening or assessment in populations with PSCD, and indirect evidence, which pertains to barriers or technology-enabling studies from broader populations with stroke or neurological disorders. Barriers were extracted, coded, and categorized into thematic domains (workforce and training challenges, health care and infrastructure, sociocultural and awareness, and logistical and economic factors). Studies focusing primarily on stroke screening technologies were included whether they involved pediatric patients with SCD or broader populations with stroke. We included them because they demonstrated scalability, feasibility, or potential applicability as future screening tools for PSCD and interpreted them in terms of implementation and accessibility characteristics rather than as direct clinical validation in populations with pediatric SCD. Finally, we grouped stroke screening technology domains into 5 higher-level categories (nonimaging, imaging-based, light-based tools, biomarkers, and AI- and ML-based mHealth solutions).

### Certainty of Evidence

Certainty of evidence was not assessed using grading of recommendations assessment, development, and evaluation (GRADE) or similar frameworks because the included studies were highly heterogeneous. We included descriptive outcomes rather than quantitative effect measures. As recommended for narrative syntheses, we provide contextual interpretation of findings rather than formal certainty ratings.

### Ethical Considerations

Institutional review board approval was not required because the study did not involve human participant research. Zotero was used for screening and managing large volumes of literature while considering transparency and accountability, bias and fairness, privacy and confidentiality, as well as validity and reliability through continuous monitoring and the application of human judgment at each step.

### Applicability of CHERRIES Guidelines

This study did not involve online surveys or web-based data collection. All analyzed data were obtained from previously published peer-reviewed studies, and 1 clearly identified preprint study was found through database searches. Therefore, the CHERRIES (Checklist for Reporting Results of Internet E-Surveys) guidelines are not applicable to this review.

## Results

### Overview

**Figure 2.**
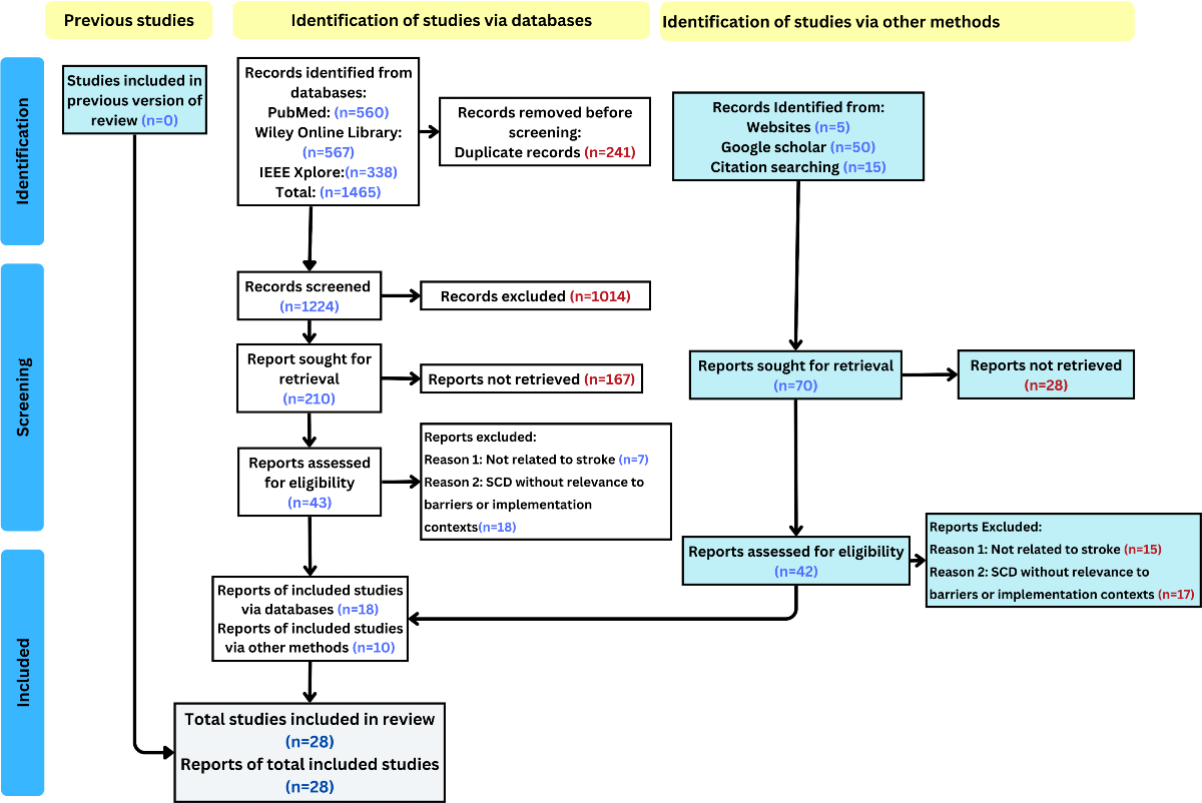
PRISMA (Preferred Reporting Items for Systematic Reviews and Meta-Analyses) flowchart illustrating the selection of the included studies and the inclusion and exclusion criteria. SCD: sickle cell disease.

Initially, a total of 1465 studies were retrieved. After excluding 241 duplicates, 1224 studies remained. During the initial screening stage, 1014 studies were excluded after reviewing the study titles and abstracts. The full texts of the remaining 85 studies were reviewed, and 57 studies were excluded based on the eligibility criteria. Finally, 28 studies were included in the systematic review. The final selection was recorded in a PRISMA flowchart in Figure 2.

### Study Characteristics

The 28 studies included in this review were conducted across diverse global regions (Multimedia Appendix 2). The largest number of studies was conducted in the United States (n=7, 25%) [11,15,20,42-45], followed by Nigeria (n=4, 14%) [46-49], India (n=4, 14%) [50-53], broader sub-Saharan Africa (n=2, 7%) [54,55], and China (n=2, 7%) [56,57]. Additional studies were conducted in Uganda (n=1, 4%) [14], Congo and Zambia (n=1, 4%) [58], Europe (n=1, 4%) [59], the Dominican Republic (n=1, 4%) [60], Taiwan (n=1, 4%) [18], Malaysia (n=1, 4%) [61], Iraq (n=1, 4%) [21], and globally (n=1, 4%) [62]. The publication years spanned from 2021 to 2025, with most studies (n=16, 57%) published between 2024 and 2025 [18,20,21,43-46,51-54,58-60,62,63], reflecting rapidly emerging research on stroke screening technologies and accessibility. Most studies were published in 2024 (n=9, 32%) [18,20,44-46,51-53,59], followed by 2025 (n=7, 25%) [21,43,54,58,60,62,63], 2022 (n=5, 18%) [14,48-50,55], 2021 (n=5, 18%) [11,42,47,56,61], and 2023 (n=2, 7%) [15,57]. Earlier foundational studies from 2021 to 2022 represented 10 (36%) of the included works [11,14,42,47-50,55,56,61].

Regarding research design, the studies used a wide range of methodologies, including qualitative descriptive studies (n=2, 7%) [42,46], retrospective observational analyses (n=4, 14%) [11,15,44,58], retrospective cohort studies (n=2, 7%) [18,43], narrative reviews (n=3, 11%) [54,56,62], cross-sectional studies (n=2, 7%) [14,59], mixed methods design and evaluation studies (n=2, 7%) [48,63], quantitative observational studies (n=2, 7%) [47,49], cluster randomized controlled trial (n=1, 4%) [61], the SACRED (Stroke Avoidance for Children in República Dominicana) trial (n=1, 4%) [60], an observational comparative study (n=1, 4%) [20], a systematic review (n=1, 4%) [55], and feasibility or technology evaluation studies (n=8, 29%) [21,44,45,50-53,57]. The focus of the studies also varied substantially, with a large proportion (n=10, 35%) centered on stroke screening accessibility and implementation challenges in LRS in both LMICs and HICs for children with sickle cell anemia (SCA) [42,43,46-49,54,55,59,60]. Additionally, 11 (39%) studies focused on novel or emerging stroke screening technologies, including MRI or MRA, optical and laser spectroscopy, blood biomarkers, AI-enhanced electrocardiogram (ECG), mobile apps, and AI-enabled multimodal tools [11,14,15,18,20,21,44,45,50-53,56-58,61-63]. Across these studies, population types ranged from children with SCA, caregivers, patients with stroke, healthy volunteers, and health care providers, allowing for a broad understanding of both accessibility barriers and technological advancements.

### RoB Assessment

Based on the assigned tools and corresponding assessments, the overall RoB across the 28 included studies showed substantial variation by study design (Multimedia Appendix 1). Of the included studies, 43% (12/28) were assessed as having low RoB. A moderate RoB was identified in 32% (9/28) of the studies, primarily among retrospective observational, cross-sectional, mixed methods, and observational study designs. In contrast, a high RoB was observed in 25% (7/28) of the studies, especially among feasibility and technology-based diagnostic accuracy studies assessed with QUADAS-2 and PROBAST, as well as more complex trial designs such as cluster randomized controlled trials. The distribution of RoB assessments across different study types and tools is summarized in Multimedia Appendix 1. As this review used narrative synthesis rather than quantitative meta-analysis, statistical assessments of reporting bias were not applicable. We qualitatively evaluated whether studies selectively reported outcomes or omitted key information. Several included studies provided limited or no details about performance, training requirements, or implementation challenges, suggesting a potential risk of reporting bias. Additionally, feasibility and technology development studies frequently emphasized only positive findings without fully reporting negative results. These limitations may lead to an incomplete representation of screening tool performance across settings.

### Barrier Characteristics

#### Overview

Using narrative synthesis, barriers were grouped into 4 domains: workforce and training constraints, health care system and infrastructure barriers, sociocultural and awareness factors, and economic and logistical constraints. The synthesized major barriers to stroke screening accessibility in LRS are presented in Multimedia Appendix 3. Of the included studies, 36% (10/28) reported barriers related to stroke screening accessibility and its implementation in different health care settings. Table 2 provides an overview of how all identified barrier categories mapped onto 4 major thematic domains: workforce and training constraints, health care system and infrastructure barriers, sociocultural and awareness factors, and economic and logistical constraints.

**Table 2.** Major categories and their corresponding barrier categories.

Major category	Barrier categories included
1. Workforce and training constraints	Lack of trained staff, lack of specialists, lack of TCD^a^ sonographers, lack of operator training, costly training, high patient volume, low provider knowledge or lack of knowledge, dependence on external examiners or visiting researchers, and limited clinical workforce capacity
2. Health care system and infrastructure barriers	Inadequate infrastructure, lack of machines or insufficient equipment, inefficient infrastructure, inefficient scheduling, coordination of appointments or fragmented care, no electronic medical records, screening services only in academic hospitals, limitations in hydroxyurea initiation or monitoring capacity, infrastructure gaps for sustained monitoring, poor health system organization, restricted TCD service availability, weak hospital leadership and partnerships, and limited neurological evaluation access
3. Sociocultural and awareness factors	Lack of awareness, cultural misconceptions, cultural disbelief, social burden, linguistic barriers, low understanding of SCA^b^ or stroke prevention, need for community engagement, fear or distrust of medical interventions, caregiver education gaps, and low health literacy.
4. Economic and logistical constraints	Costly treatment, high cost of TCD, MRA^c^, or CBT^d^, transportation issues, logistical difficulties, high lifestyle demands, low funding, socioeconomic treatment variation, travel burdens, competing domestic responsibilities, geographic inaccessibility, out-of-pocket expenses for care, appointment scheduling conflicts, and caregiver time constraints.

a TCD: transcranial Doppler.

b SCA: sickle cell anemia.

c MRA: magnetic resonance angiography.

d CBT: chronic blood transfusion therapy.

#### Workforce and Training Challenges

As depicted in Multimedia Appendix 3, the lack of trained staff was reported in 60% (6/10) of the studies [46,47,54,55,59,60]. These studies indicated that stroke screening methods such as Doppler ultrasound and TCD require specialized training for operators, which is one of the major barriers to performing TCD screening in many LMICs. Several studies mentioned the need to train a specialized team to operate TCD, while others suggested training existing physicians and nurses to conduct the screening method, thereby reducing the need for specialists. From the 10 studies, 1 (10%) reported a lack of specialists [48] and 1 (10%) reported a lack of TCD sonographers [49].

#### Health Care Systems and Infrastructure

Thirty percent (3/10) of the studies highlighted inadequate infrastructure [47,54,55], and 20% (2/10) reported complex health care as a major health care system and infrastructure-related barrier [43,60] (Multimedia Appendix 3). Many rural and remote areas lack adequate infrastructure to perform screening procedures due to lack of equipment, poor coordination of scheduling, and a lack of laboratories to perform required tests. Additionally, follow-up care is inconsistent due to transportation difficulties and health care system inefficiencies, even when screenings are completed [42,48].

#### Sociocultural and Awareness

Forty percent (4/10) of the studies reported that a lack of awareness among patients and health care providers indicates that their knowledge of the importance of stroke screening is significantly low [46,55,59,60] (Multimedia Appendix 3). Health care providers are not aware of the annual TCD screening, and their lack of knowledge about stroke symptoms and risk factors further results in delayed diagnosis and treatment. Most of the time, caregivers are also not aware of the importance of early detection and risk factors, which diminishes the effectiveness of screening programs. Additionally, 40% (4/10) of the studies revealed that social burden and cultural disbelief contribute to reduced stroke screening assessments [46,49,54,55].

#### Economic and Logistical Challenges

Fifty percent (5/10) of the studies identified costly treatment as a primary barrier [43,47,49,55,60] (Multimedia Appendix 3). From the 10 studies, 1 (10%) revealed costly operator training [48], 1 (10%) revealed costly TCD examination [49], and 1 (10%) revealed costly blood transfusion therapy [49]. Additionally, 1 (10%) study reported that many public health care systems lack the funding essential to subsidize these costs [59]. In some LMICs, the cost of screening depends on patients’ personal expenses, further limiting accessibility.

### Disparities of Stroke Screening Access in LRS, HICs, and LMICs

From Multimedia Appendix 3, a clear disparity exists in stroke screening accessibility across low-resource environments in both LMICs and LRS in HICs. Evidence from LMICs shows that shortages of trained personnel, limited availability of TCD machines, fragile health care infrastructure, and high out-of-pocket costs consistently restrict early stroke detection among children with SCA. Caregivers also face substantial financial and social burdens, along with inadequate awareness of stroke risks and limited community outreach mechanisms in LMICs. Infrastructure challenges, such as shortages of electronic medical records, insufficient specialists, and dependence on external partnerships, hinder the accessibility of stroke screening. Similarly, LRS within HICs demonstrated parallel barriers; however, they mostly face logistical difficulties in scheduling and coordinating care in already established health care facilities. Other significant barriers in HICs include transportation limitations and high lifestyle demands that interfere with attending appointments. Socioeconomic disadvantages are strong in HICs and are strongly associated with inconsistent access to screening, reduced follow-up, and poorer biological risk markers, reflecting systemic inequities despite comparatively advanced health systems. On the other hand, LMICs face widespread difficulties in accessing stroke screening facilities. In both contexts, limited trained staff, operational inefficiencies, and socioeconomic barriers are major concerns regarding reduced early stroke prevention.

### Existing Core and Emerging Stroke Screening Technology Characteristics

Narrative synthesis from 64% (18/28) of the included studies revealed 5 distinct categories of stroke screening technologies [11,14,15,18,20,21,44,45,50-53,56-58,61-63] (Multimedia Appendix 4). At a higher level, we could categorize as follows: (1) nonimaging technology-based tools, such as TCD, (2) imaging-based modalities, such as MRI and MRA, (3) light-based optical spectroscopy-based devices, including SCOS and laser-based approaches, (4) biomarker-based and physiological signal-based approaches, such as routine blood test-derived markers, and (5) AI- and ML-enabled mHealth and wearable tools. These technologies could overlap with each other for stroke screening purposes. The last 3 categories could be considered emerging technologies as they are still progressing toward validation.

### Existing Technologies

Of the included studies, 7% (2/28) reported that nonimaging TCD is suitable for early-age screening and has a low technical failure rate but is highly specialized and operator-dependent [11,58]. Similarly, 7% (2/28) of the studies focused on magnetic field-based imaging screening, such as MRI and MRA stroke screening tools, which produce high sensitivity and detailed cerebral imaging. However, these tools require radiologists to perform and interpret the results [14,15] (Multimedia Appendix 4). They are limited by high cost, inadequate infrastructure, and portability issues and are recommended only for children in serious condition.

### Emerging Technologies

Light-based optical spectroscopy devices are among the emerging stroke screening technologies in 7% (2/28) of the studies. These devices are lightweight and require only basic instructions [20,21]. Similarly, 7% (2/28) of the studies examined blood biomarker-based stroke screening, whose main advantage is that it can be conducted using clinically accessible, routine blood test data [18,44]. AI and ML were used in 29% (8/28) of the studies to enhance mobile and wearable apps, as well as to improve blood biomarkers-based stroke detection or risk prediction [18,45,50,52,53,56,62,63]. These technologies showed promise in terms of accessibility, real-time analysis, and scalability. Wearable-based technologies for stroke screening were reported in 11% (3/28) of the studies [56,57,62], while 18% (5/28) examined mobile app–based stroke screening tools [45,51,52,61,63]. In addition, ECG-based cerebral blood flow monitoring devices were evaluated in 4% (1/28) of the studies and demonstrated strong performance in real-time monitoring and stroke prediction [50].

### Cost, Portability, Scalability, and Infrastructure Needs

Low-cost stroke screening methods were reported in 25% (7/28) of the studies [11,20,21,44,56,58,61]. Among these studies, 7% (2/28) involved TCD-based stroke screening [11,58], 7% (2/28) examined light-based optical devices [20,21], 4% (1/28) examined blood biomarker-based screening methods [44], 4% (1/28) evaluated mobile-based solutions [61], and 4% (1/28) evaluated wearable solutions [56], which were considered highly suitable for LRS. In contrast, 7% (2/28) of the studies mentioned MRI or MRA-based techniques as high-cost [14,15], and these imaging technologies require significant infrastructure and trained specialists, making scalability challenging in LRS. Furthermore, 7% (2/28) of the studies are highly portable as they are lightweight, light-based imaging technologies [20,21]. Finally, 36% (10/28) of the studies show potential for highly scalable tools, including mobile-based technologies [45,51,52,61,63], wearable-based technologies [56,57,62], and biomarker tests [18,44], which are easily deployable in LRS due to their minimal to no infrastructure requirements.

### Training Requirements

High dependency on the machine operator was mentioned in 14% (4/28) of the studies [11,14,15,58]. Emerging technologies such as light-based optical spectroscopy, blood biomarkers, mobile-based tools, AI-based systems, and wearables were reported in 50% (14/28) of the studies as being less operator-dependent and easily adoptable in LRS [18,20,21,44,45,50-53,56,57,61-63]. AI tools that are easily integrated with various devices and require little to no technical training were explored in 22% (6/28) of the studies, making them ideal for self-use or by basic clinical staff [18,45,52,53,62,63]. Mobile app devices and decision support systems, providing a new way for self-management of stroke screening, were reported in 18% (5/28) of the studies [45,51,52,61,63].

### Outcomes and Challenges

Wearable-based studies showing potential for high scalability in the future were reported in 11% (3/28) of the studies [56,57,62]. AI- and ML-based studies showing an accuracy of around 90% were identified in 22% (6/28) of the studies [18,45,50,53,62,63]. An AI- and ECG-based stroke screening solution model with more than 90% accuracy was developed in 4% (1/28) of the studies [50]. An ML-based mobile app reporting around 88% user satisfaction during use was described in 4% (1/28) of the studies [63]. A multimodal mobile app demonstrating an accuracy of 80.25% was reported in 4% (1/28) of the studies [45] and 7% (2/28) of the studies focused on the implementation of TCD [11,58], which mentioned that TCD has a low technical failure rate of around 4.3% but encountered problems due to implementation challenges.

### mHealth Solution From Identified Barriers and Technological Tools Characteristics

**Figure 3.**
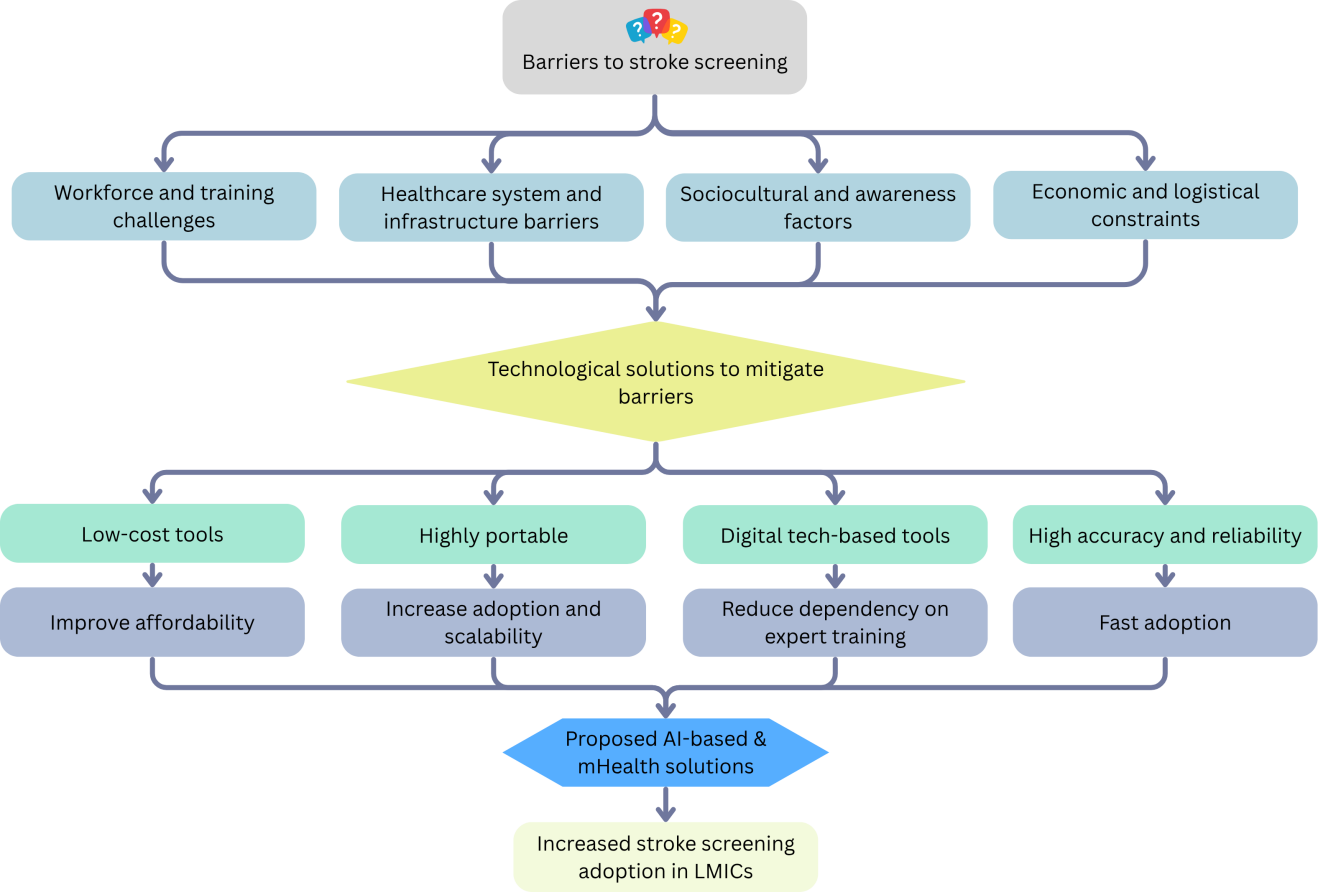
Conceptual framework integrating the key barriers identified in research question (RQ) 1 with the benefits and addressing the limitations of stroke screening technologies from RQ2, aiming to develop an innovative mobile health (mHealth)–enabled solution for low-resource settings. AI: artificial intelligence; LMIC: low- and middle-income country.

We integrate our findings from RQ1 and RQ2 to build a conceptual framework that illustrates how various barriers in LRS impact stroke screening accessibility and how different aspects of technological solutions for stroke screening can lead to an innovative approach that increases the adoption of stroke screening technologies in LMICs. The framework is illustrated in Figure 3.

**Figure 4.**
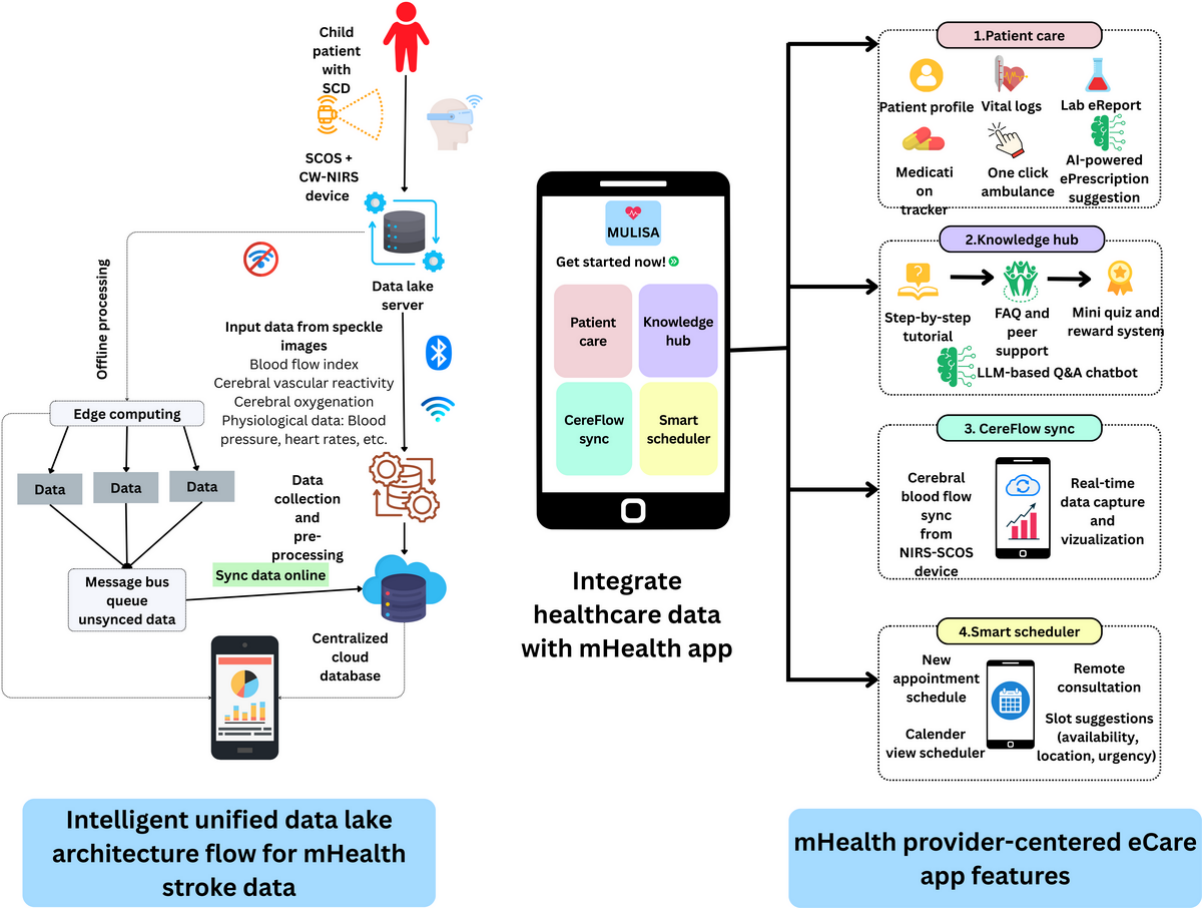
Modular unified lightweight intelligent stroke assistant (MULISA) mobile health (mHealth) system architecture consists of 5 major modules (data lake for raw data inputs, data processing unit, centralized server, artificial intelligence [AI] data analyzer, and main application module) and 3 supporting modules. FAQ: frequently asked questions; LLM: large language model; SCD: sickle cell disease; SCOS: speckle contrast optical spectroscopy; CW-NIRS: continuous wave–near-infrared spectroscopy.

This section addresses RQ3 by proposing a modular mHealth system specifically tailored to support stroke screening and care management in children with SCD. The system embraces a user-centered approach, emphasizing real-world health care provider workflows, intuitive mobile interfaces, and accessibility in low-resource environments. By analyzing barriers identified from RQ1 and examining stroke screening technologies from RQ2, we suggest an end-to-end solution for stroke screening and assessment for SCD among pediatric patients. This solution will integrate a mobile app with dual-wavelength SCOS and continuous wave near-infrared spectroscopy (NIRS) devices, which will measure cerebral blood flow and oxygenation noninvasively, providing real-time, actionable insights about stroke risk into the portable mobile dashboard. This system will process real-time data, provide interactive clinical guidance and remote consultations, and enable health care providers to quickly assess a patient’s condition by viewing user-friendly dashboards, allowing them to respond and provide care more easily [64]. Additionally, the solution will incorporate edge computing, making it accessible even in offline environments and ensuring stability in LRS. Overall, this innovative approach will ensure continuous monitoring, early intervention, and enhanced clinical decision-making, ultimately improving health outcomes for children with SCD. Figure 4 illustrates our novel mHealth system architecture designed to facilitate stroke screening and patient management, integrated with an NIRS-SCOS stroke screening tool. These features will be co-designed in accordance with user-centered guidelines [65], ensuring that the interface and data visualizations are both understandable and actionable, even for nonspecialist providers.

## Discussion

### Principal Findings

This systematic review examined the multilevel barriers to PSCD stroke screening (RQ1), summarized existing and emerging screening technologies (RQ2), and explored opportunities for user-centered mHealth integration to address accessibility gaps in low-resource environments (RQ3).

Narrative synthesis allowed us to integrate diverse evidence and identify core barrier themes across settings, highlighting structural constraints and workflow gaps that must be addressed through integrated mHealth solutions. This review identified substantial barriers in stroke screening accessibility for patients with PSCD across LRS, driven largely by workforce challenges, limited infrastructure, awareness, and financial constraints (RQ1; Multimedia Appendix 3). Across LMICs and HICs, we found consistent themes related to a lack of staff, inadequate infrastructure, lack of knowledge, and financial constraints. These findings align with earlier regional studies reporting that TCD uptake remains low due to limited trained sonographers, insufficient radiology capacity, and fragmented care pathways [54,55]. It is also evident that economic limitations are among the most significant barriers in LMICs [49,55]. Procedures like TCD, MRA, and chronic blood transfusion therapy are efficient stroke prevention methods but are often out of reach due to high costs [11,14,49]. Even when TCD equipment was implemented, services could not be scaled due to a lack of lab facilities, a proper appointment scheduling structure, and insufficient operational resources [47,54]. These studies also reveal that some programs fail to achieve sustainability despite initial investments due to systemic weaknesses in health care infrastructure and coordination. In HICs, cost is not reported as a dominant factor; however, operational and logistical inefficiencies, such as scheduling conflicts, limited radiology hours, and poor integration of stroke screening tools, affect screening adherence [42,43]. Cultural beliefs, socioeconomic factors, and low awareness about stroke prevention among patients’ primary caregivers and health care providers reduce the possibilities of taking preventive measures in LRS in both LMICs and HICs [43,46,49,54,60]. Most people fear long-term dependency on medical treatments like blood transfusions, leading them to avoid taking medical help in the early stages of the disease [49].

To answer RQ2, we explored screening technologies and found out 5 major categories of stroke screening technologies (imaging, nonimaging, biomarkers, SCOS-based tools, and mHealth or AI solutions), each demonstrating unique strengths but varying widely in accessibility and feasibility. Furthermore, an exploration of both existing and emerging technologies related to stroke screening (Multimedia Appendix 4) highlights critical attributes, including cost, portability, scalability, infrastructure needs, training requirements, and outcomes. It reveals the significant potential of AI-driven mHealth solutions for the early diagnosis of stroke risk by analyzing the key characteristics of each stroke screening tool, particularly in resource-limited settings, and the flexibility of customizing mHealth solutions for patients with PSCD [45,52,53,63].

Finally, our findings underscore the need for an integrated, user-centered mHealth solution capable of responding to the identified barriers and leveraging the key functional attributes of emerging stroke screening tools for early stroke detection in patients with PSCD (RQ3). The potential impact of these barriers and screening tools, introduced in Figure 4, will help bridge gaps in early diagnosis, empower local health care workers, and promote timely follow-up care for critical conditions such as SCD. As this solution will be mHealth-based, it will be low-cost, highly portable, and scalable in LRS. A knowledge hub module will be incorporated to address the lack of knowledge and inadequate operator training in stroke screening assessments. This module will aid in decision-making by providing step-by-step instructions, a peer support system, and a large language model–based chatbot. AI will be used to provide advanced analytics on patient risk assessment reports by analyzing historical data [66]. User engagement will be promoted through the app, where we will design and assess the app’s interactive dashboard built from collected stroke screening data from the SCOS device [67], support remote consultation, and include SCD-specific care recommendations, such as hydration tracking and pain episode logs [68]. Furthermore, the system’s mobile-first architecture (using a Flutter frontend and Django backend) will ensure cross-platform compatibility and offline functionality, key advantages for deployment in rural or infrastructure-challenged areas. The whole solution will be incorporated with edge computing so that it will have consistent connectivity even in offline environments, which is a major requirement in LRS. Future field testing will involve pilot studies to evaluate usability, adoption rates, clinical effectiveness, and user satisfaction, to make this a real-world tool. Overall, this research demonstrates a promising path forward for scalable, user-centered mHealth solutions that can significantly improve pediatric stroke risk management in resource-limited settings.

### Limitations

This review has several methodological limitations. There may be language and publication bias, as only English-language publications were included. Additionally, the exclusion of gray literature, conference abstracts, and non–English-language publications could mean that we have omitted valuable regional or local insights, especially from non–English-speaking LMICs, where relevant work might be found. Additionally, valuable information from other sources that are not indexed in major databases may be overlooked. Although the review followed PRISMA guidelines, screening and data extraction were independently conducted by two reviewers to minimize selection and interpretation bias. However, small differences in interpretation are likely to occur naturally.

This systematic review aimed to capture the most up-to-date stroke screening technologies; however, it has certain limitations. One of the primary concerns is the heterogeneity of the studies included in terms of their methodologies, the technologies evaluated, and the outcome measures used, which prevented us from performing a meta-analysis. As a result, this review was unable to determine which stroke screening technology is most closely linked to effective interventions.

Another notable limitation is related to the rapidly evolving and growing nature of artificial intelligence and mHealth technologies. Some of the most recent developments may not yet be available in peer-reviewed literature and, therefore, were excluded from our analysis. Furthermore, many studies focus on technical performance rather than evaluating real-world deployment, usability, and long-term sustainability.

### Future Directions

There is a clear need for further research to understand the barriers present in LRS involving large populations. Emerging technologies like AI-based mobile apps stand out for their low cost and high portability, making them highly adoptable and scalable in LRS. Integrating mobile-based apps with stroke screening devices will increase the ability to detect unknown stroke signs early. Integrating AI with imaging technologies may offer future pathways to overcome some of the prevailing infrastructure and cost barriers, making imaging more accessible and enhancing diagnostic accuracy. Furthermore, additional research is needed to refine and validate emerging stroke screening technologies. Future work should also focus on improving the generalizability of these tools across diverse populations and different stroke severities, as well as enhancing the integration of these technologies with existing health care systems. There is also potential for multimodal AI systems to provide a more comprehensive and accurate risk assessment for strokes, as well as the integration of mHealth systems with emerging technologies such as NIRS tools or ECG tools.

### Conclusions

In this review, the authors have identified various key barriers limiting access to stroke screening for children with SCD in LRS and evaluated the sustainability of both standard and emerging technological solutions. This review revealed a wide array of prevailing tools that remain inaccessible due to several economic, cultural, and infrastructural barriers commonly encountered in LRS, as well as emerging stroke screening technologies that could be easily used in various health care settings. Additionally, this review demonstrated potential solutions for stroke screening and assessment, underscoring the promise of integrating mHealth solutions with portable devices and imaging systems. While significant advancements have been made in the development of stroke screening technologies, there still remains a critical challenge to ensure that these tools are accessible, accurate, and scalable in real-world settings. Finally, we uncovered an AI-enabled solution customized to address the identified barriers. This holistic solution offers a practical foundation to revolutionize stroke prevention and management, guide clinical implementation, and drive health innovation in the future, particularly in resource-limited environments.

## Supplementary material

10.2196/76937 Checklist 1 PRISMA checklist.

10.2196/76937 Multimedia Appendix 1 Risk-of-bias assessment of included studies.

10.2196/76937 Multimedia Appendix 2 Study characteristics.

10.2196/76937 Multimedia Appendix 3 Barriers to stroke screening accessibility.

10.2196/76937 Multimedia Appendix 4 Stroke screening technologies.

## Abbreviations

AI: artificial intelligence
CHERRIES: Checklist for Reporting Results of Internet E-Surveys
ECG: electrocardiogram
GRADE: Grading of Recommendations Assessment, Development and Evaluation
HIC: high-income country
JBI: Joanna Briggs Institute
LMIC: low- and middle-income country
LRS: low-resource settings
MeSH: Medical Subject Headings
mHealth: mobile health
ML: machine learning
MMAT: Mixed Methods Appraisal Tool
MRA: magnetic resonance angiography
MRI: magnetic resonance imaging
NIRS: near-infrared spectroscopy
PICO: population, intervention, comparison, and outcome
PRISMA: Preferred Reporting Items for Systematic Reviews and Meta-Analyses
PROBAST: Prediction Model Risk of Bias Assessment Tool
PSCD: pediatric sickle cell disease
QUADAS-2: Quality Assessment of Diagnostic Accuracy Studies–2
RoB: risk-of-bias
RQ: research question
SACRED: Stroke Avoidance for Children in República Dominicana
SCA: sickle cell anemia
SCD: sickle cell disease
SCOS: speckle contrast optical spectroscopy
TCD: transcranial Doppler
