# Supplementary material for: mHealth-Enabled Stroke Screening for Pediatric Sickle Cell Disease in Low-Resource Settings: Systematic Literature Review of Critical Barriers, Emerging Technologies, and AI-Driven Solutions
Source: JMIR Pediatr Parent. 2026 Apr 6;9:e76937. doi: 10.2196/76937 (PMC13053000; doi:10.2196/76937)
Supplement: Multimedia Appendix 1 [file pediatrics-v9-e76937-s002.docx]

**Risk of Bias Assessment of Included Studies**

**Risk of bias assessment summary:**

| **Study No.** | **Study Design** | **Assigned RoB Tool** | **Overall RoB** |
| --- | --- | --- | --- |
| ^1^ | Qualitative Descriptive | **JBI Qualitative** | **Low** |
| ^2^ | Narrative Review | **JBI Text & Opinion** | **Low** |
| ^3^ | Quantitative Descriptive | **JBI Analytical Cross-Sectional** | **Moderate** |
| ^4^ | Mixed Methods | **MMAT** | **Moderate** |
| ^5^ | Quantitative Observational | **JBI Analytical Cross-Sectional** | **Moderate** |
| ^6^ | Systematic Review | **JBI Systematic Review & Research Syntheses** | **Low** |
| ^7^ | Qualitative Descriptive | **JBI Qualitative** | **Low** |
| ^8^ | Retrospective Cohort | **Newcastle-Ottawa Scale (NOS) Cohort** | **Low** |
| ^9^ | Randomized Controlled Trial (SACRED trial RCT) | **RoB 2.0 (SACRED trial)** | **High** |
| ^10^ | Cross-Sectional Survey | **JBI Analytical Cross-Sectional** | **Moderate** |
| ^11^ | Retrospective Observational | **Newcastle-Ottawa Scale (NOS) Cohort** | **Moderate** |
| ^12^ | Retrospective Observational | **Newcastle-Ottawa Scale (NOS) Cohort** | **Moderate** |
| ^13^ | Retrospective Observational | **Newcastle-Ottawa Scale (NOS) Cohort** | **Moderate** |
| ^14^ | Cross-Sectional | **JBI Analytical Cross-Sectional** | **Low** |
| ^15^ | Feasibility (Technology) | **QUADAS-2** | **High** |
| ^16^ | Observational Comparative | **QUADAS-2** | **Moderate** |
| ^17^ | Retrospective Observational | **Newcastle-Ottawa Scale (NOS) Cohort** | **Moderate** |
| ^18^ | Retrospective Cohort | **PROBAST** | **Low** |
| ^19^ | Feasibility (AI/ECG) | **PROBAST** | **High** |
| ^20^ | Feasibility | **QUADAS-2** | **High** |
| ^21^ | Cluster RCT | **RoB 2.0 (Cluster RCT)** | **High** |
| ^22^ | Feasibility (AI/ML) | **PROBAST** | **Low** |
| ^23^ | Feasibility (Mobile AI triage) | **PROBAST** | **Low** |
| ^24^ | Narrative Review | **JBI Text & Opinion** | **Low** |
| ^25^ | Feasibility (Wearable Device) | **QUADAS-2** | **High** |
| ^26^ | Feasibility (ML model) | **PROBAST** | **Low** |
| ^27^ | Mixed Methods Design and Evaluation | **PROBAST** | **High** |
| ^28^ | Narrative Review | **JBI Text & Opinion** | **Low** |

**JBI Critical Appraisal Checklist for Qualitative Research:**

| **No.** | **Criteria** | **Yes** | **No** | **Unclear** | **Not Applicable** |
| --- | --- | --- | --- | --- | --- |
| 1 | Is there congruity between the stated philosophical perspective and the research methodology? |  |  |  |  |
| 2 | Is there congruity between the research methodology and the research question or objectives? |  |  |  |  |
| 3 | Is there congruity between the research methodology and the methods used to collect data? |  |  |  |  |
| 4 | Is there congruity between the research methodology and the representation and analysis of data? |  |  |  |  |
| 5 | Is there congruity between the research methodology and the interpretation of results? |  |  |  |  |
| 6 | Is there a statement locating the researcher culturally or theoretically? |  |  |  |  |
| 7 | Is the influence of the researcher on the research, and vice versa, addressed? |  |  |  |  |
| 8 | Are participants, and their voices, adequately represented? |  |  |  |  |
| 9 | Is the research ethical according to current criteria, and is there evidence of ethical approval? |  |  |  |  |
| 10 | Do the conclusions drawn in the research report flow from the analysis or interpretation of the data? |  |  |  |  |

Risk of Bias assessment with **JBI Qualitative:**

**Yes - Y**

**No - N**

**Unclear – U**

**Not Applicable - NA**

| **Study Reference** | **Criteria** | | | | | | | | | | **Overall risk of bias** |
| --- | --- | --- | --- | --- | --- | --- | --- | --- | --- | --- | --- |
|  | 1 | 2 | 3 | 4 | 5 | 6 | 7 | 8 | 9 | 10 |  |
| 1 | Y | Y | Y | Y | Y | Y | U | Y | Y | Y | Low |
| 7 | Y | Y | Y | Y | Y | U | U | Y | Y | Y | Low |

**JBI Analytical Cross-Sectional Checklist:**

| **JBI Criterion** | **Yes** | **No** | **Unclear** | **Not Applicable** |
| --- | --- | --- | --- | --- |
| 1. Were the criteria for inclusion in the sample clearly defined? |  |  |  |  |
| 2. Were the study subjects and the setting described in detail? |  |  |  |  |
| 3. Was the exposure measured in a valid and reliable way? |  |  |  |  |
| 4. Were objective, standard criteria used for measurement of the condition? |  |  |  |  |
| 5. Were confounding factors identified? |  |  |  |  |
| 6. Were strategies to deal with confounding factors stated? |  |  |  |  |
| 7. Were the outcomes measured in a valid and reliable way? |  |  |  |  |
| 8. Was appropriate statistical analysis used? |  |  |  |  |

**Risk of Bias assessment with JBI Cross-Sectional Checklist:**

**Yes - Y**

**No - N**

**Unclear – U**

**Not Applicable - NA**

| **Study Ref** | **Criteria** | | | | | | | | **Overall RoB** |
| --- | --- | --- | --- | --- | --- | --- | --- | --- | --- |
|  | 1 | 2 | 3 | 4 | 5 | 6 | 7 | 8 |  |
| 3 | Y | Y | Y | Y | N | N | Y | Y | Moderate |
| 5 | Y | Y | Y | Y | N | N | Y | Y | Moderate |
| 10 | Y | Y | U | Y | Y | Y | Y | Y | Moderate |
| 14 | Y | Y | Y | Y | Y | Y | Y | Y | Low |

**JBI CRITICAL APPRAISAL CHECKLIST FOR TEXT AND OPINION PAPERS:**

| **JBI Criterion** | **Yes** | **No** | **Unclear** | **Not Applicable** |
| --- | --- | --- | --- | --- |
| 1. Is the source of the opinion clearly identified? |  |  |  |  |
| 2. Does the source of opinion have standing in the field of expertise? |  |  |  |  |
| 3. Are the interests of the relevant population the central focus of the opinion? |  |  |  |  |
| 4. Is the stated position the result of an analytical process, and is there logic in the opinion expressed? |  |  |  |  |
| 5. Is there reference to the extant literature? |  |  |  |  |
| 6. Is any incongruence with the literature/sources logically defended? |  |  |  |  |

**Risk of Bias assessment with JBI Text & Opinion Checklist:**

Yes – Y, No – N, Unclear – U

Not Applicable - NA

| **Study Ref** | **Criteria** | | | | | | **Overall RoB** |
| --- | --- | --- | --- | --- | --- | --- | --- |
|  | 1 | 2 | 3 | 4 | 5 | 6 |  |
| 2 | Y | Y | Y | Y | Y | Y | Low |
| 24 | Y | Y | Y | Y | Y | N | Low |
| 28 | Y | Y | Y | Y | Y | U | Low |

**JBI Systematic Review & Research Syntheses Checklist:**

| **JBI Criterion** | **Yes** | **No** | **Unclear** | **Not Applicable** |
| --- | --- | --- | --- | --- |
| **1. Is the review question clearly and explicitly stated?** |  |  |  |  |
| **2. Were the inclusion criteria appropriate for the review question?** |  |  |  |  |
| **3. Was the search strategy appropriate?** |  |  |  |  |
| **4. Were the sources and resources used to search for studies adequate?** |  |  |  |  |
| **5. Were the criteria for appraising studies appropriate?** |  |  |  |  |
| **6. Was critical appraisal conducted by two or more reviewers independently?** |  |  |  |  |
| **7. Were there methods to minimize errors in data extraction?** |  |  |  |  |
| **8. Were the methods used to combine studies appropriate?** |  |  |  |  |
| **9. Was the likelihood of publication bias assessed?** |  |  |  |  |
| **10. Were recommendations for policy and/or practice supported by the reported data?** |  |  |  |  |
| **11. Were the specific directives for new research appropriate?** |  |  |  |  |

**Risk of Bias assessment with JBI Systematic Review & Research Syntheses:**

**Yes - Y**

**No - N**

**Unclear – U**

**Not Applicable - NA**

| Study Reference | Criteria | | | | | | | | | | | Overall risk of bias |
| --- | --- | --- | --- | --- | --- | --- | --- | --- | --- | --- | --- | --- |
|  | 1 | 2 | 3 | 4 | 5 | 6 | 7 | 8 | 9 | 10 | 11 |  |
| 6 | Y | Y | Y | Y | Y | Y | Y | Y | N | Y | Y | Low |

**NEWCASTLE - OTTAWA QUALITY ASSESSMENT SCALE COHORT STUDIES Checklist:**

| Domain/Criteria | stars(*) /no stars |
| --- | --- |
| **Selection** | |
| 1. Representativeness of the exposed cohort  a) truly representative of the average _______________ (describe) in the community *  b) somewhat representative of the average ______________ in the community *  c) selected group of users eg nurses, volunteers  d) no description of the derivation of the cohort |  |
| 2. Selection of the non exposed cohort  a) drawn from the same community as the exposed cohort *  b) drawn from a different source  c) no description of the derivation of the non exposed cohort |  |
| 3. Ascertainment of exposure  a) secure record (eg surgical records) *  b) structured interview *  c) written self report  d) no description |  |
| 4) Demonstration that outcome of interest was not present at start of study  a) yes *  b) no |  |
| **Comparability** | |
| 1) Comparability of cohorts on the basis of the design or analysis  a) study controls for _____________ (select the most important factor) *  b) study controls for any additional factor * (This criteria could be modified to indicate specific control for a second important factor.) |  |
| **Outcome** | |
| 1. Assessment of outcome  a) independent blind assessment *  b) record linkage *  c) self report  d) no description |  |
| 2. Was follow-up long enough for outcomes to occur  a) yes (select an adequate follow up period for outcome of interest) *  b) no |  |
| 3. Adequacy of follow up of cohorts  a) complete follow up - all subjects accounted for *  b) subjects lost to follow up unlikely to introduce bias - small number lost - > ____ % (select an  adequate %) follow up, or description provided of those lost) 8  c) follow up rate < ____% (select an adequate %) and no description of those lost  d) no statement |  |

**Risk of Bias assessment with Newcastle-Ottawa Scale (NOS):**

**Note:** A study can be awarded a maximum of one star for each numbered item within the Selection and Outcome categories. A maximum of two stars can be given for Comparability

Highest Rating means Low Risk of Bias

Selection - S

Comparability - C

Outcome – O

| Study Ref | Criteria | | | | | | | | | | Overall RoB |
| --- | --- | --- | --- | --- | --- | --- | --- | --- | --- | --- | --- |
|  | S1 | S2 | S3 | S4 | C1a | C1b | O1 | O2 | O3 | NOS Score (out of 9) |  |
| 8 | * | * | * | * | * | * | * | * | * | 9 | Low |
| 11 | * | * | * |  |  |  | * | * | * | 6 | Moderate |
| 12 | * | * | * |  | * |  | * | * | * | 7 | Moderate |
| 13 |  | * | * | * |  |  | * | * | * | 6 | Moderate |
| 17 | * | * | * | * | * | * | * |  |  | 7 | Moderate |

**QUADAS-2 Checklist:**

| Domain/Question | Yes/No/Unclear |
| --- | --- |
| **DOMAIN 1: PATIENT SELECTION** | |
| 1. Was a consecutive or random sample of patients enrolled? |  |
| 2. Was a case-control design avoided? |  |
| 3. Did the study avoid inappropriate exclusions? |  |
| **DOMAIN 2: INDEX TEST(S)** | |
| 4. Were the index test results interpreted without knowledge of the results of the reference standard? |  |
| 5. If a threshold was used, was it pre-specified? |  |
| **DOMAIN 3: REFERENCE STANDARD** | |
| 6. Is the reference standard likely to correctly classify the target condition? |  |
| 7. Were the reference standard results interpreted without knowledge of the results of the index test? |  |
| **DOMAIN 4: FLOW AND TIMING** | |
| 8. Was there an appropriate interval between index tests and reference standard? |  |
| 9. Did all patients receive a reference standard? |  |
| 10.Did all patients receive the same reference standard? |  |
| 11. Were all patients included in the analysis? |  |

**Risk of Bias assessment with QUADAS-2:**

Yes – Y, No – N, Unclear – U, Not Applicable - NA

High – H, Low – L, Moderate - M

Overall RoB for Domain 1: ORD1

Overall RoB for Domain 2: ORD2

Overall RoB for Domain 3: ORD3

Overall RoB for Domain 4: ORD4

| **Study Ref** | **Criteria** | | | | | | | | | | | | | | | **Overall RoB** |
| --- | --- | --- | --- | --- | --- | --- | --- | --- | --- | --- | --- | --- | --- | --- | --- | --- |
|  | 1 | 2 | 3 | ORD1 | 4 | 5 | ORD2 | 6 | 7 | ORD3 | 8 | 9 | 10 | 11 | ORD4 |  |
| 16 | N | N | N | H | U | U | U | Y | Y | L | Y | Y | Y | N | L | Moderate |
| 20 | N | U | U | H | U | U | H | Y | U | H | U | Y | Y | Y | L | High |
| 25 | U | Y | N | H | U | N | H | Y | U | M | Y | Y | Y | Y | L | High |

**PROBAST Checklist:**

| **Domain/Question** | **Yes/No/Unclear** |
| --- | --- |
| **DOMAIN 1: Participants** | |
| 1.1 Were appropriate data sources used, e.g. cohort, RCT or nested case control study data? |  |
| 1.2 Were all inclusions and exclusions of participants appropriate? |  |
| A. Risk of Bias: Risk of bias introduced by selection of participants | RISK: (low/ high/ unclear) |
| B. Applicability: Concern that the included participants and setting do not match the review question | CONCERN: (low/ high/ unclear) |
| **DOMAIN 2: Predictors** | |
| 2.1 Were predictors defined and assessed in a similar way for all participants? |  |
| 2.2 Were predictor assessments made without knowledge of outcome data? |  |
| 2.3 Are all predictors available at the time the model is intended to be used? |  |
| A. Risk of Bias: Risk of bias introduced by predictors or their assessment | RISK: (low/ high/ unclear) |
| B. Applicability: Concern that the definition, assessment or timing of predictors in the model do not match the review question | CONCERN: (low/ high/ unclear) |
| **DOMAIN 3: Outcome** | |
| 3.1 Was the outcome determined appropriately? |  |
| 3.2 Was a pre-specified or standard outcome definition used? |  |
| 3.3 Were predictors excluded from the outcome definition? |  |
| 3.4 Was the outcome defined and determined in a similar way for all participants? |  |
| 3.5 Was the outcome determined without knowledge of predictor information? |  |
| 3.6 Was the time interval between predictor assessment and outcome determination appropriate? |  |
| A. Risk of Bias: Risk of bias introduced by the outcome or its determination | RISK: (low/ high/ unclear) |
| B. Applicability: Concern that the outcome, its definition, timing or determination do not match the review question | CONCERN: (low/ high/ unclear) |
| **DOMAIN 4: Analysis** | |
| 4.1 Were there a reasonable number of participants with the outcome? |  |
| 4.2 Were continuous and categorical predictors handled appropriately? |  |
| 4.3 Were all enrolled participants included in the analysis? |  |
| 4.4 Were participants with missing data handled appropriately? |  |
| 4.5 Was selection of predictors based on univariable analysis avoided? |  |
| 4.6 Were complexities in the data (e.g. censoring, competing risks, sampling of controls) accounted for appropriately? |  |
| 4.7 Were relevant model performance measures evaluated appropriately? |  |
| 4.8 Were model overfitting and optimism in model performance accounted for? |  |
| 4.9 Do predictors and their assigned weights in the final model correspond to the results from multivariable analysis? |  |
| A. Risk of Bias: Risk of bias introduced by the analysis | RISK: (low/ high/ unclear) |

**Risk of Bias assessment with PROBAST:**

Yes (Y), Probably yes (PY), Probably no (PN), No (N) or no information (NI)

High – H, Low – L, Unclear - U

Domain 1: D1

Domain 2: D2

Domain 3: D3

Domain 4: D4

| **Study Ref** | **Criteria** | | | | | | | | | | | | | | | | | | | | | | | | | | | | **Overall RoB** | |
| --- | --- | --- | --- | --- | --- | --- | --- | --- | --- | --- | --- | --- | --- | --- | --- | --- | --- | --- | --- | --- | --- | --- | --- | --- | --- | --- | --- | --- | --- | --- |
|  | D1 | | | | D2 | | | | | D3 | | | | | | | | D4 | | | | | | | | | |  | |  |
|  | 1 | 2 | A | B | 1 | 2 | 2 | A | B | 1 | 2 | 3 | 4 | 5 | 6 | A | B | 1 | 2 | 3 | 4 | 5 | 6 | 7 | 8 | 9 | A |  |  |  |
| 18 | Y | U | H | M | Y | Y | Y | L | L | Y | Y | Y | Y | Y | Y | L | L | Y | Y | N | Y | N | U | Y | Y | Y | L | L | |  |
| 19 | Y | N | H | M | Y | U | Y | L | L | Y | U | Y | U | U | Y | U | M | Y | U | U | Y | Y | U | Y | N | N | H | H | |  |
| 22 | Y | U | L | U | Y | U | Y | L | L | U | U | Y | U | Y | U | H | H | Y | Y | U | U | Y | U | Y | N | Y | L | L | |  |
| 23 | Y | Y | L | L | Y | Y | Y | L | L | Y | Y | Y | Y | U | Y | L | L | Y | Y | N | N | Y | Y | Y | N | Y | M | L | |  |
| 26 | Y | U | U | H | Y | Y | Y | L | L | Y | U | U | U | U | U | H | H | Y | Y | U | Y | Y | Y | Y | U | U | L | L | |  |
| 27 | U | U | H | H | Y | U | Y | L | L | U | U | Y | U | U | U | H | H | N | Y | N | N | Y | Y | N | N | N | H | H | |  |

**RoB 2 CRT Checklist:**

| Domain/Question | Y / PY / PN / N / NI |
| --- | --- |
| **Domain 1a: Risk of bias arising from the randomization process** | |
| 1a.1 Was the allocation sequence random? |  |
| 1a.2 Was the allocation sequence concealed until clusters were enrolled and assigned to interventions? |  |
| 1a.3 Did baseline differences between intervention groups suggest a problem with the randomization process? |  |
| Risk-of-bias judgement | Low/ High/ Some concerns |
| **Domain 1b: Risk of bias arising from the timing of identification or recruitment of participants in a cluster-randomized trial** | |
| 1b.1 Were all the individual participants identified and recruited (if appropriate) before randomization of clusters? |  |
| 1b.2 If N/PN/NI to 1b.1: Is it likely that selection of individual participants was affected by knowledge of the intervention assigned to the cluster? | NA/Y/PY/PN/N/NI |
| 1b.3 Were there baseline imbalances that suggest differential identification or recruitment of individual participants between intervention groups? |  |
| Risk-of-bias judgement | Low/ High/ Some concerns |
| **Domain 2: Risk of bias due to deviations from the intended interventions (effect of assignment to intervention)** | |
| 2.1a Were participants aware that they were in a trial? |  |
| 2.1b. If Y/PY/NI to 2.1a: Were participants aware of their assigned intervention during the trial? | NA / Y / PY / PN / N / NI |
| 2.2. Were carers and people delivering the interventions aware of participants assigned intervention during the trial? |  |
| 2.3. If Y/PY/NI to 2.1 or 2.2: Were there deviations from the intended intervention that arose because of the trial context? | NA / Y / PY / PN / N / NI |
| 2.4 If Y/PY to 2.3: Were these deviations likely to have affected the outcome? | NA / Y / PY / PN / N / NI |
| 2.5. If Y/PY/NI to 2.4: Were these deviations from intended intervention  balanced between groups? | NA / Y / PY / PN / N / NI |
| 2.6 Was an appropriate analysis used to estimate the effect of assignment to intervention? |  |
| 2.7 If N/PN/NI to 2.6: Was there potential for a substantial impact (on the result) of the failure to analyse participants in the group to which they were randomized? | NA / Y / PY / PN / N / NI |
| Risk-of-bias judgement | Low/ High/ Some concerns |
| **Domain 2: Risk of bias due to deviations from the intended interventions (effect of adhering to intervention)** | |
| 2.1. Were participants aware of their assigned intervention during the trial? |  |
| 2.2. Were carers and people delivering the interventions aware of participants assigned intervention during the trial? |  |
| 2.3. [If applicable:] If Y/PY/NI to 2.1 or 2.2: Were important non-protocol interventions balanced across intervention groups? | NA / Y / PY / PN / N / NI |
| 2.4. [If applicable:] Were there failures inimplementing the intervention that could have affected the outcome? | NA / Y / PY / PN / N / NI |
| 2.5. [If applicable:] Was there non-adherence to the assigned intervention regimen that could have affected participants’ outcomes? | NA / Y / PY / PN / N / NI |
| 2.6. If N/PN/NI to 2.3, or Y/PY/NI to 2.4 or 2.5: Was an appropriate analysis used to estimate the effect of adhering to the intervention? | NA / Y / PY / PN / N / NI |
| Risk-of-bias judgement | Low/ High/ Some concerns |
| **Domain 3: Risk of bias due to missing outcome data** | |
| 3.1a Were data for this outcome available for all clusters that recruited participants? |  |
| 3.1b Were data for this outcome available for all, or nearly all, participants within clusters? |  |
| 3.2 If N/PN/NI to 3.1a or 3.1b: Is there evidence that the result was not biased by missing data? | NA / Y / PY / PN / N / NI |
| 3.3 If N/PN to 3.2 Could missingness in the outcome depend on its true value? | NA / Y / PY / PN / N / NI |
| 3.4 If Y/PY/NI to 3.3: Is it likely that  missingness in the outcome depended on  its true value? | NA / Y / PY / PN / N / NI |
| Risk-of-bias judgement | Low/ High/ Some concerns |
| **Domain 4: Risk of bias in measurement of the outcome** | |
| 4.1 Was the method of measuring the outcome inappropriate? |  |
| 4.2 Could measurement or ascertainment of the outcome have differed between intervention groups? |  |
| 4.3a If N/PN/NI to 4.1 and 4.2: Were outcome assessors aware that a trial was taking place? | NA / Y / PY / PN / N / NI |
| 4.3b If Y/PY/NI to 4.3a: Were outcome assessors aware of the intervention received by study participants? | NA / Y / PY / PN / N / NI |
| 4.4 If Y/PY/NI to 4.3b: Could assessment of the outcome have been influenced by knowledge of intervention received? | NA / Y / PY / PN / N / NI |
| 4.5 If Y/PY/NI to 4.4: Is it likely that assessment of the outcome was influenced by knowledge of intervention received? | NA / Y / PY / PN / N / NI |
| Risk-of-bias judgement | Low/ High/ Some concerns |

**RoB 2 CRT Assessments:**

Yes (Y), Probably yes (PY), Probably no (PN), No (N) or no information (NI)

RoB Judgment – J

Low – L

High- H

Some Concerns – C

| **Study Ref** | **Criteria** | | | | | | | | | | | | | | | | | | | | | | | | | | **Overall RoB** |
| --- | --- | --- | --- | --- | --- | --- | --- | --- | --- | --- | --- | --- | --- | --- | --- | --- | --- | --- | --- | --- | --- | --- | --- | --- | --- | --- | --- |
|  | D1a | | | | D1b | | | | D2 | | | | | | | | D3 | | | | | D4 | | | | |  |
|  | 1 | 2 | 3 | J | 1 | 2 | 3 | J | 1 | 2 | 3 | 4 | 5 | 6 | 7 | J | 1 | 2 | 3 | 4 | J | 1 | 2 | 3 | 4 | 5 |  |
| 9 | N | N | N | H | N | Y | N | H | N | N | Y | N | N | Y | N | H | Y | Y | N | N | H | Y | Y | N | N | H | H |
| 21 | Y | Y | N | C | N | Y | N | H | Y | Y | Y | NA | NA | Y | NA | C | Y | NI | NI | NI | C | N | N | N | N | N | H |

**MMAT Checklist:**

| **Category of study designs** | **Methodological quality criteria** | **Responses (Yes / No / Can’t tell)** |
| --- | --- | --- |
| **Screening questions (for all types)** | S1. Are there clear research questions? |  |
|  | S2. Do the collected data allow to address the research questions? |  |
| Note: Further appraisal may not be feasible or appropriate when the answer is ‘No’ or ‘Can’t tell’ to one or both screening questions. | | |
| **1. Qualitative** | 1.1. Is the qualitative approach appropriate to answer the research question? |  |
|  | 1.2. Are the qualitative data collection methods adequate to address the research question? |  |
|  | 1.3. Are the findings adequately derived from the data? |  |
|  | 1.4. Is the interpretation of results sufficiently substantiated by data? |  |
|  | 1.5. Is there coherence between qualitative data sources, collection, analysis and interpretation? |  |
| **2. Quantitative randomized controlled trials** | 2.1. Is randomization appropriately performed? |  |
|  | 2.2. Are the groups comparable at baseline? |  |
|  | 2.3. Are there complete outcome data? |  |
|  | 2.4. Are outcome assessors blinded to the intervention provided? |  |
|  | 2.5. Did the participants adhere to the assigned intervention? |  |
| **3. Quantitative non-randomized** | 3.1. Are the participants representative of the target population? |  |
|  | 3.2. Are measurements appropriate regarding both the outcome and intervention (or exposure)? |  |
|  | 3.3. Are there complete outcome data? |  |
|  | 3.4. Are the confounders accounted for in the design and analysis? |  |
|  | 3.5. During the study period, is the intervention administered (or exposure occurred) as intended? |  |
| **4. Quantitative descriptive** | 4.1. Is the sampling strategy relevant to address the research question? |  |
|  | 4.2. Is the sample representative of the target population? |  |
|  | 4.3. Are the measurements appropriate? |  |
|  | 4.4. Is the risk of nonresponse bias low? |  |
|  | 4.5. Is the statistical analysis appropriate to answer the research question? |  |
| **5. Mixed methods** | 5.1. Is there an adequate rationale for using a mixed methods design to address the research question? |  |
|  | 5.2. Are the different components of the study effectively integrated to answer the research question? |  |
|  | 5.3. Are the outputs of the integration of qualitative and quantitative components adequately interpreted? |  |
|  | 5.4. Are divergences and inconsistencies between quantitative and qualitative results adequately addressed? |  |
|  | 5.5. Do the different components of the study adhere to the quality criteria of each tradition of the methods involved? |  |

**MMAT Assessment:**

High – H, Moderate – M, Low - L

Can’t tell: C

| Study Ref | Criteria | | | | | | | | | | | | | | | | | | | | | | | | | | | Overall Rob |
| --- | --- | --- | --- | --- | --- | --- | --- | --- | --- | --- | --- | --- | --- | --- | --- | --- | --- | --- | --- | --- | --- | --- | --- | --- | --- | --- | --- | --- |
|  | S | | 1 | | | | | 2 | | | | | 3 | | | | | 4 | | | | | 5 | | | | |  |
|  | 1 | 2 | 1 | 2 | 3 | 4 | 5 | 1 | 2 | 3 | 4 | 5 | 1 | 2 | 3 | 4 | 5 | 1 | 2 | 3 | 4 | 5 | 1 | 2 | 3 | 4 | 5 |  |
| 4 | Y | Y | Y | Y | C | C | Y | NA | | | | | Y | Y | C | Y | Y | NA | | | | | Y | Y | C | C | Y | M |

**References:**

1. Bello-Manga H, Haliru L, Ahmed K, et al. Barriers and facilitators to a task-shifted stroke prevention program for children with sickle cell anemia in a community hospital: a qualitative study. *Implement Sci Commun*. 2024;5(1). doi:10.1186/s43058-023-00534-z

2. Latham TS, Czabanowska K, Babich S, Yego-Kosgei F, Shook LM, Ware RE. Primary Stroke Screening and Hydroxyurea Treatment for Sickle Cell Anemia in Pediatric Healthcare Settings in East and Central Africa: A Narrative Review of Capacity Gaps and Opportunities. *Public Health Rev*.*Frontiers Media SA*. 2025;46. doi:10.3389/phrs.2025.1608359

3. Ghafuri DL, Covert Greene B, Musa B, et al. Capacity Building for Primary Stroke Prevention Teams in Children Living With Sickle Cell Anemia in Africa. *Pediatr Neurol*. 2021;125:9-15. doi:10.1016/j.pediatrneurol.2021.08.010

4. Bello-Manga H, Haliru L, Ahmed KA, et al. Primary Prevention of Stroke in Children with Sickle Cell Anemia in Nigeria: Protocol for a Mixed Methods Implementation Study in a Community Hospital. *JMIR Res Protoc*. 2022;11(6). doi:10.2196/37927

5. Ghafuri DL, Abdullahi SU, Dambatta AH, et al. *Establishing Sickle Cell Disease Stroke Prevention Teams in Africa Is Feasible: Program Evaluation Using the RE-AIM Framework*. Vol 44.; 2022. www.jpho-online.com

6. Mwangi LW, Abuga JA, Cottrell E, Kariuki SM, Kinyanjui SM, Newton CRJC. Barriers to access and utilization of healthcare by children with neurological impairments and disability in low-and middle-income countries: a systematic review. *Wellcome Open Res*. 2021;6:61. doi:10.12688/wellcomeopenres.16593.1

7. Phillips SM, Schlenz AM, Mueller M, Melvin CL, Adams RJ, Kanter J. Identified barriers and facilitators to stroke risk screening in children with sickle cell anemia: results from the DISPLACE consortium. *Implement Sci Commun*. 2021;2(1). doi:10.1186/s43058-021-00192-z

8. Edwards JG, Humphrey DT, Mueller M, et al. Socioeconomic Status and Stroke Risk in Pediatric Sickle Cell Disease: A DISPLACE Study Secondary Analysis. *Pediatr Blood Cancer*. Published online October 13, 2025. doi:10.1002/pbc.32112

9. Nieves RM, Latham T, Marte N, et al. Stroke prevention in Hispanic children with sickle cell anemia: the SACRED trial. *Blood Adv*. 2025;9(8):1791-1800. doi:10.1182/bloodadvances.2024014327

10. Voi V, Gutierrez-Valle V, Cuzzubbo D, et al. Limited access to transcranial Doppler screening and stroke prevention for children with sickle cell disease in Europe: Results of a multinational EuroBloodNet survey. *Pediatr Blood Cancer*. 2024;71(10). doi:10.1002/pbc.31190

11. Kanter J, Phillips S, Schlenz AM, et al. Transcranial Doppler Screening in a Current Cohort of Children with Sickle Cell Anemia: Results from the DISPLACE Study. *J Pediatr Hematol Oncol*. 2021;43(8):E1062-E1068. doi:10.1097/MPH.0000000000002103

12. Tembo D, Moons P, Tshimanga T, et al. Optimizing Transcranial Doppler Screening for Stroke Prevention in Young African Children With Sickle Cell Disease. *Pediatr Blood Cancer*. 2025;72(9). doi:10.1002/pbc.31861

13. Strumph K, Morrone K, Dhillon P, et al. Impact of magnetic resonance angiography parameters on stroke prevention therapy in pediatric patients with sickle cell anemia. *Pediatr Blood Cancer*. 2023;70(2). doi:10.1002/pbc.30109

14. Idro R, Boehme AK, Kawooya M, et al. Brain Magnetic Resonance Imaging and Angiography in Children with Sickle Cell Anaemia in Uganda in a Cross-Sectional Sample. *Journal of Stroke and Cerebrovascular Diseases*. 2022;31(4). doi:10.1016/j.jstrokecerebrovasdis.2022.106343

15. Taha BA, Kadhim AC, Addie AJ, et al. Optical Spectroscopy of Cerebral Blood Flow for Tissue Interrogation in Ischemic Stroke Diagnosis. *ACS Chem Neurosci*. 2025;16(5):895-907. doi:10.1021/acschemneuro.4c00809

16. Huang YX, Mahler S, Abedi A, et al. Correlating stroke risk with non-invasive cerebrovascular perfusion dynamics using a portable speckle contrast optical spectroscopy laser device. *Biomed Opt Express*. 2024;15(10):6083. doi:10.1364/boe.534796

17. Nri-Ezedi CA, Efobi CC, Campbell A. Diagnostic Potential of Platelet-Neutrophil Ratio (PNR) for Stroke Risk in Sickle Cell Anaemia Children Authors and Affiliations: Preprint posted online July 12, 2024. doi:10.22541/au.172081281.17980876/v1

18. Bamodu OA, Chan L, Wu CH, Yu SF, Chung CC. Beyond diagnosis: Leveraging routine blood and urine biomarkers to predict severity and functional outcome in acute ischemic stroke. *Heliyon*. 2024;10(4). doi:10.1016/j.heliyon.2024.e26199

19. Anand Kumar M, Abirami N, Guru Prasad MS, Mohankumar M. Stroke Disease Prediction based on ECG Signals using Deep Learning Techniques. In: *Proceedings of International Conference on Computational Intelligence and Sustainable Engineering Solution, CISES 2022*. Institute of Electrical and Electronics Engineers Inc.; 2022:453-458. doi:10.1109/CISES54857.2022.9844403

20. Vasa S, Savani S, Thaker A, Bhangale P. StrokeAlert: Early Detection of Minor Strokes. In: *2024 4th International Conference on Advancement in Electronics and Communication Engineering, AECE 2024*. Institute of Electrical and Electronics Engineers Inc.; 2024:168-172. doi:10.1109/AECE62803.2024.10911730

21. Mat Said Z, Musa KI, Tengku Ismail TA, et al. The Effectiveness of Stroke RiskometerTM in Improving Stroke Risk Awareness in Malaysia: A Study Protocol of a Cluster-Randomized Controlled Trial. *Neuroepidemiology*. 2021;55(6):436-446. doi:10.1159/000518853

22. Shah M, Sahasranaman K, Sondur C, Nadagire R, Bhatia G. CDSS based Mobile Application for Stroke Assistance. In: *Proceedings - 2024 2nd International Conference on Inventive Computing and Informatics, ICICI 2024*. Institute of Electrical and Electronics Engineers Inc.; 2024:439-446. doi:10.1109/ICICI62254.2024.00078

23. Cai T, Wong K, Wang JZ, et al. M3 Stroke: Multi-Modal Mobile AI for Emergency Triage of Mild to Moderate Acute Strokes. In: *BHI 2024 - IEEE-EMBS International Conference on Biomedical and Health Informatics, Proceedings*. Institute of Electrical and Electronics Engineers Inc.; 2024. doi:10.1109/BHI62660.2024.10913652

24. Chen YH, Sawan M. Trends and challenges of wearable multimodal technologies for stroke risk prediction. *Sensors (Switzerland)*.*MDPI AG*. 2021;21(2):1-23. doi:10.3390/s21020460

25. Zeng L, Li G, Xu J, et al. A Wearable Device for Dynamic Monitoring of Cerebral Blood Flow Based on Electromagnetic Coupling Sensing. *IEEE Sens J*. 2023;23(23):29571-29584. doi:10.1109/JSEN.2023.3324309

26. Sharma M, Kaur I, Saini G, Thakur V, Mishra A. Predictive Modeling for Stroke Risk Assessment Using Machine Learning. In: *2024 International Conference on Intelligent Systems for Cybersecurity, ISCS 2024*. Institute of Electrical and Electronics Engineers Inc.; 2024. doi:10.1109/ISCS61804.2024.10581155

27. Yusro M, Syam R, Idrus A, Rikawarastuti, Apriyansa A. A Machine Learning-Based Mobile Health Application for Early Stroke Risk Prediction and Prevention. In: Institute of Electrical and Electronics Engineers (IEEE); 2025:1-7. doi:10.1109/iccit65724.2025.11167669

28. Olawade DB, Aderinto N, Clement David-Olawade A, et al. Integrating AI-driven wearable devices and biometric data into stroke risk assessment: A review of opportunities and challenges. *Clin Neurol Neurosurg*.*Elsevier B.V.* 2025;249. doi:10.1016/j.clineuro.2024.108689
