# Supplementary material for: mHealth-Enabled Stroke Screening for Pediatric Sickle Cell Disease in Low-Resource Settings: Systematic Literature Review of Critical Barriers, Emerging Technologies, and AI-Driven Solutions
Source: JMIR Pediatr Parent. 2026 Apr 6;9:e76937. doi: 10.2196/76937 (PMC13053000; doi:10.2196/76937)
Supplement: Multimedia Appendix 2 [file pediatrics-v9-e76937-s003.docx]

**Study Characteristics**

| **Author** | **Study Year** | **Country** | **Study Design** | **Sample Size** | **Population Type** |
| --- | --- | --- | --- | --- | --- |
| (Bello-Manga et al., 2024) | 2024 | Nigeria | Qualitative Descriptive | 2 focus groups + 5 interviews | Children with SCA |
| (Latham et al., 2025) | 2025 | East & Central Africa (multiple countries) | Narrative Review | 37 Publications | Children with SCA |
| (Ghafuri et al., 2021) | 2021 | Nigeria | Quantitative Descriptive | 679 children, 23 trained healthcare personnel | Children with SCA |
| (Bello-Manga et al., 2022) | 2022 | Nigeria | Mixed Methods | Not reported sample | Children with SCA |
| (Ghafuri et al., 2022) | 2022 | Nigeria | Quantitative Observational | 3200 screened children in 5 hospitals | Children with SCA |
| (Mwangi et al., 2022) | 2022 | Sub-Saharan Africa | Systematic Review | 20 Studies | Children with neurological impairments in LMICs |
| (Phillips et al., 2021) | 2021 | United States | Qualitative Descriptive | 52 semi-structured interviews | Patients with SCA, caregivers, and healthcare providers |
| (Edwards et al., 2025) | 2025 | United States | Retrospective Cohort | 3124 Children | Children with SCD (ages 2-6) with at least one TCD screen |
| (Nieves et al., 2025) | 2025 | Dominican Republic | Randomized Controlled Trial (SACRED trial RCT) | 283 Children | Hispanic children with SCA |
| (Voi et al., 2024) | 2024 | European countries | Cross-Sectional | 81 experts from 77 healthcare centers across 16 countries | Healthcare providers, children with SCD |
| (Kanter et al., 2021) | 2021 | USA | Retrospective Observational | 5247 children | Children with SCA |
| (Tembo et al., 2025) | 2025 | DR Congo & Zambia | Retrospective Observational | 115 children | Young children with SCD |
| (Strumph et al., 2023) | 2023 | USA | Retrospective Observational | 21 pediatric patients | Pediatric SCA patients |
| (Idro et al., 2022) | 2022 | Uganda | Cross-sectional | 81 children | Children with SCA |
| (Taha et al., 2025) | 2025 | Iraq | Feasibility (technology) | Simulations + clinical tests | Stroke patients (acute ischemic) |
| (Huang et al., 2024) | 2024 | USA | Observational Comparative | 53 participants | Stroke Patients (low vs high stroke risk groups) |
| (Nri-Ezedi et al., 2024) | 2024 | USA | Retrospective observational | 144 children | SCD HbSS children |
| (Bamodu et al., 2024) | 2024 | Taiwan | Retrospective Cohort | 2229 patients | patients with acute ischemic stroke (AIS) |
| (Anand Kumar et al., 2022) | 2022 | India | Feasibility (AI/ECG) | 4068 records of ECG samples | Stroke Patients |
| (Vasa et al., 2024) | 2024 | India | Feasibility | Test Dataset:  Facial Drooping  Detection:960  Speech Difficulty  Evaluation: 300  Arm Weakness detection: 50 | Healthy individuals + Stroke Patients |
| (Mat Said et al., 2021) | 2021 | Malaysia | Cluster RCT | Two groups, 66 in each group | One with the app, another with standard management |
| (Shah et al., 2024) | 2024 | India | Feasibility (AI/ML) | 2 stroke prediction datasets: 5110 and 4798 samples | Individuals with health-related factors |
| (Cai et al., 2024) | 2024 | USA | Feasibility (Mobile AI triage) | 269 participants | Mild-moderate stroke patients |
| (Chen & Sawan, 2021) | 2021 | China | Narrative review | N/A | Wearable tech users |
| (Zeng et al., 2023) | 2023 | China | Feasibility (Wearable Device) | 24 healthy volunteers | Potential  stroke individuals and stroke patients |
| (Sharma et al., 2024) | 2024 | India | Feasibility (ML model) | 3926 training + 982 testing patient records | Stroke Patients |
| (Yusro et al., 2025) | 2025 | Indonesia | Mixed-methods Design and Evaluation | Not mentioned, Two distinct user groups | Younger adults and  elderly users |
| (Olawade et al., 2025) | 2025 | Global | Narrative review | Not reported the number of papers | Stroke & wearable tech studies |
