# Supplementary material for: mHealth-Enabled Stroke Screening for Pediatric Sickle Cell Disease in Low-Resource Settings: Systematic Literature Review of Critical Barriers, Emerging Technologies, and AI-Driven Solutions
Source: JMIR Pediatr Parent. 2026 Apr 6;9:e76937. doi: 10.2196/76937 (PMC13053000; doi:10.2196/76937)
Supplement: Multimedia Appendix 3 [file pediatrics-v9-e76937-s004.docx]

**Barriers to Stroke Screening Accessibility**

| **Study** | **Study Year** | **Country** | **Study Design** | **Sample Size** | **Population Type** | **Relevance to PSCD Stroke Screening** | **Country Income Level (World Bank)** | **Resource Context (System-Level)** | **Stroke Screening Accessibility Challenges** | **Barriers Category** |
| --- | --- | --- | --- | --- | --- | --- | --- | --- | --- | --- |
| (Bello-Manga et al., 2024) | 2024 | Nigeria | Qualitative Descriptive | 2 focus groups + 5 interviews | Children with SCA | Direct | LMIC | Low-resource setting | 1. Insufficient staffing due to low pay, high turnover  2. Not everyone can be trained to perform task-shifted TCD screening.  3. Patients parents facing financial and social burdens that impact their ability to access and utilize the stroke screening program.  4. Need to provide appropriate information about the stroke prevention program to both healthcare providers and caregivers of children with SCA.  5. Facilitating caregiver buy-in by delivering education and awareness in a consistent and convenient manner, such as during regular SCD clinic visits.  6. Engaging community members as "champions" to help increase awareness and reach of the TCD screening program.  7. Utilizing community health workers to create awareness about SCA and the TCD screening program among the broader community. | 1. Lack of Trained Staff  2. Lack of Awareness  3. High cost of treatment  4. Social Burden |
| (Latham et al., 2025) | 2025 | East & Central Africa (multiple countries) | Narrative Review | 37 Publications | Children with SCA | Direct | LMIC | Low-resource setting | 1. Lack of skilled personnel trained to administer TCD exams  2. Lack of understanding of SCA at the provider and patient/family level in sub-Saharan Africa  3. Inadequate healthcare infrastructure to provide continuity of care across the lifespan | 1. Lack of Trained Staff  2. Inadequate Infrastructure  3. Lack of Knowledge  4. Cultural Misconception |
| (Ghafuri et al., 2021) | 2021 | Nigeria | Quantitative Descriptive | 679 children, 23 trained healthcare personnel | Children with SCA | Direct | LMIC | Low-resource setting | 1. Lack of research experience in low-resource setting  2. One pediatric neurologist in the region  3. No electronic medical records  4. Lack of TCD machines and trained operators  5. Hydroxyurea not affordable for many families outside of the trial | 1. Lack of Trained Staff  2. Inadequate Infrastructure  3. Lack of Operator Training  4. Lack of Machines  5. Logistical Difficulties  6. Costly Treatment |
| (Bello-Manga et al., 2022) | 2022 | Nigeria | Mixed Methods | Not reported sample | Children with SCA | Direct | LMIC | Low-resource setting | 1. High cost associated with the multiple training courses that nurses and medical officers have to undergo  2. Stroke prevention programs are only available at academic hospitals where there are many specialists that offer these services. | 1. Costly Training  2. Lack of Specialists  3. Transportation Issues |
| (Ghafuri et al., 2022) | 2022 | Nigeria | Qualitative Observational | 3200 screened children in 5 hospitals | Children with SCA | Direct | LMIC | Low-resource setting | 1. Number of children with sickle cell anemia (SCA) is higher than the Screening Programs.  2. The scarcity of TCD machines in the region posed a significant barrier.  3. Guardians rejected treatment due to the regional practice of requiring families to regularly seek blood donors  4. High cost of red blood cell units relative to income.  5. Comparatively high charge for performing TCD examination  6. Scarcity of TCD-certified sonographers  7. Slow development of state hospital leaders, and partnerships to activate the Memorandum of Understanding.  8. Costly blood transfusion therapy | 1. High Volume of Patients  2. Insufficient Equipment  3. Cultural disbelief  4. Costly TCD examination  5. Lack of TCD sonographers  6. Social Burden  7. Costly Treatment |
| (Mwangi et al., 2022) | 2022 | Sub-Saharan Africa | Systematic Review | 20 Studies | Children with neurological impairments in LMICs | Indirect | LMIC | Low-resource setting | Financial constraints  Geographical and physical inaccessibility  Inadequate healthcare resources  Cultural and belief-related barriers  Lack of education/awareness  Competing domestic responsibilities  Issues with privacy/confidentiality | 1. Lack of Trained Staff  2. Lack of Awareness  3. Costly Treatment  4. Social Burden  5. Inadequate Infrastructure |
| (Phillips et al., 2021) | 2021 | United States | Qualitative Descriptive | 52 semi-structured interviews | Patients with SCA, caregivers, and healthcare providers | Direct | HIC | Low-resource within HIC | 1. High life demands of patients and caregivers  2. Need for providers to track missed appointments  3. Organizational capacity to manage rescheduling effectively.  4.Complicated processes for scheduling TCD screenings separately from clinic visits  5. Healthcare providers reported logistical challenges of scheduling, transportation, and the overall organization of care.  6. Work and school commitments, often conflicted with the ability to attend appointments. | 1. High Volume of Patients  2. Inefficient Scheduling  3. Coordination Appointments  4. Logistical Difficulties  5. High Lifestyle Demands  6. Lack of Staff |
| (Edwards et al., 2025) | 2025 | United States | Retrospective Cohort | 3124 Children | Children with SCD (ages 2-6) with at least one TCD screen | Direct | HIC | Low-resource within HIC | 1. Lack of HU prescription was the strongest factor associated with abnormal TCD results.  2. Children living in “Very Low” Childhood Opportunity Index (COI) neighborhoods showed socioeconomic status-linked challenges, including inconsistent care access.  3. socioeconomic status-related barriers such as transportation, follow-up difficulty, schedule conflicts, and caregiver resource limitations likely impact TCD completion indirectly.  4. Lower-socioeconomic status groups displayed worse biological markers (lower hemoglobin, higher WBC), factors associated with increased stroke risk. | 1. Complex Healthcare  2. Costly Treatment  3. High Lifestyle Demands  4. Socioeconomic Treatment Variation |
| (Nieves et al., 2025) | 2025 | Dominican Republic | Randomized Controlled Trial (SACRED trial RCT) | 283 Children | Hispanic children with SCA | Direct | UMIC  (Upper-Middle-Income) | Low-resource within UMIC | 1. TCD is currently limited because of the cost and availability of screening  2. Local healthcare system lacked certified TCD examiners  3. Limited capacity for hydroxyurea initiation, dose escalation, and long-term monitoring.  4. Children presented with severe anemia and high TCD velocities due to delayed diagnosis and lack of early screening.  5. MRI revealed substantial baseline parenchymal disease, indicating delayed access to neurological evaluation.  6. Infrastructure gaps for sustained monitoring  7. Dependence on external partnership to establish TCD program reflects limited local resources and training pathways | 1. Lack of Trained Staff  2. Costly Treatment  3. Lack of Awareness  4. Linguistic Barriers  5. Inadequate Infrastructure  6. Complex Healthcare |
| (Voi et al., 2024) | 2024 | European countries | Cross-Sectional Survey | 81 experts from 77 healthcare centers across 16 countries | Healthcare providers, children with SCD | Direct | HIC | Low-resource within HIC | 1. Lack of dedicated TCD/TCDi service  for children.  2. Lack of trained staff to perform TCD screenings  3. Refusal of patients due to logistical difficulties like TCD in another City  4. Lack of funding for dedicated staff and equipment  5.Expert centers having TCD facilities, patients are sent to other hospitals to perform TCD due to lack of trained staff.  6.Lack of awareness and education among healthcare professionals, specifically hematologists and pediatric hematologists. | 1. Logistical Difficulties  2. Lack of Trained Staff  3. Low Funding  4. Lack of Awareness |
