# Supplementary material for: mHealth-Enabled Stroke Screening for Pediatric Sickle Cell Disease in Low-Resource Settings: Systematic Literature Review of Critical Barriers, Emerging Technologies, and AI-Driven Solutions
Source: JMIR Pediatr Parent. 2026 Apr 6;9:e76937. doi: 10.2196/76937 (PMC13053000; doi:10.2196/76937)
Supplement: Multimedia Appendix 4 [file pediatrics-v9-e76937-s005.docx]

**Stroke Screening Technologies**

| **Study Ref** | **Year** | **Country/Region** | **Study Design** | **Sample Size** | **Population Type** | **Relevance to PSCD Stroke Screening** | **Tool Type** | **Tool Characteristics** | **Training Requirements** | **Outcome** | **Challenges** |
| --- | --- | --- | --- | --- | --- | --- | --- | --- | --- | --- | --- |
| (Kanter et al., 2021) | 2021 | USA | Retrospective Observational | 5247 children | Children with SCA | Direct | Non-Imaging  (TCD) | Cost: Moderate, Portable, Moderate, Scalability | Requires certified sonographer | Highly accurate, gold standard for pediatric stroke risk | Limited availability, personnel shortage |
| (Tembo et al., 2025) | 2025 | DR Congo & Zambia | Retrospective Observational | 115 children | Young children with SCD | Direct | Non-Imaging  (TCD) | Early-age screening, Non-invasive, identifies early cerebral vasculopathy  Cost: Inexpensive, Portable | Highly operator dependent | Low technical failure rate (4.3%) | Refine guidelines needed for early-age TCD screening.  Need improved methods to reduce agitation related failure |
| (Strumph et al., 2023) | 2023 | USA | Retrospective Observational | 21 pediatric patients | Pediatric SCA patients | Direct | Imaging-MRI MRA | Optimized MRA scanning using the SWiTCH protocol by reducing TE to <5 ms, upgrading MRI from 1.5T to 3T, and increasing voxel size for better SNR, Low scalability | Interpretation of MRA  imaging dependent on neuroradiologist. | 50% had vasculopathy downgraded with the new MRA, correcting overdiagnoses, avoiding unnecessary transfusions, and enabling better treatment decisions | Radiologists interpretation may vary.  Linking MR protocol changes to treatment decisions is challenging. |
| (Idro et al., 2022) | 2022 | Uganda | Cross-Sectional | 81 children | Children with SCA | Direct | MRI and MRA | Detected imaging abnormalities and severe vessel narrowing in children. Expensive, Low portability, Poor scalability | Radiologists, technicians, neurocognitive testing team needed | Highly sensitive in detecting silent infarcts and stenosis missed by TCD | Small sample size, single-site sample limits generalizability.  Lacks correlation between imaging abnormalities. |
| (Taha et al., 2025) | 2025 | Iraq | Feasibility (technology) | Simulations + clinical tests | Stroke patients (acute ischemic) | Indirect | Optical Spectroscopy based Technology | - Detects cerebral blood flow changes - Low Cost - Portable laser tool | Provides rapid feedback, ideal for bedside clinical use. | The 976 nm wavelength is highly sensitive to ischemia | Improve SCOS models to boost accuracy and reduce errors.  Integration with imaging systems will enhance stroke detection |
| (Huang et al., 2024) | 2024 | USA | Observational Comparative | 53 participants | Stroke Patients (low vs high stroke risk groups) | Indirect | Speckle Contrast Optical Spectroscopy (SCOS) | Uses a laser diode and CMOS camera to non-invasively track brain blood flow which reveals physiological insights for stroke risk assessment.   - Lightweight, Portable, Cost-effective, Non-invasive | Need basic instructions | Low-risk group showed greater brain blood volume increases; high-risk group had higher blood flow.  BHICBF/BHICBV ratio differences (p = 0.000001) highlight SCOS’s potential to assess cerebrovascular health | Small subgroup size (6–10) limits statistical conclusions.  Future work: Assess validation for long-term outcomes in stroke prevention |
| (Nri-Ezedi et al., 2024) (Preprint) | 2024 | USA | Retrospective Observational | 144 children | SCD HbSS children | Direct | Blood Biomarkers | Used platelet-neutrophil ratio (PNR) for stroke risk: 83.83 normal, 75.69 conditional, 62.57 abnormal (p = 0.031).  Cost-effective, easily accessible. | Clinicians trained in routine blood tests can interpret this | Multivariate analysis showed higher PNR linked to lower odds of abnormal TCD (OR = 0.975, p = 0.0345) | Clinical validation needed, promising TCD alternative in resource-limited settings. |
| (Bamodu et al., 2024) | 2024 | Taiwan | Retrospective Cohort | 2229 patients | patients with acute ischemic stroke (AIS) | Indirect | Blood Biomarkers | common clinical biomarkers and ML to assess stroke risk, offering transparent, interpretable predictions without needing extra data like age or sex. | Clinically accessible and routinely tested blood data. | XGBoost showed lowest RMSE, identifying key biomarkers and effectively predicting stroke severity and outcomes | single-center, moderate sample size, and only baseline biomarkers used, missing changes over stroke stages or treatment response |
| (Anand Kumar et al., 2022) | 2022 | India | Feasibility (AI/ECG) | 4068 records of ECG samples | Stroke Patients | Indirect | AI-Based ECG | - A medical framework for detecting ECG abnormalities related to stroke. - Deep learning using LSTM to detect stroke-related diseases from ECG, EEG, blood pressure, pulse, and pedometer data | Improve health professional efficiency in quick diagnosis. | Accuracy: 93.78% | Potential for better disease diagnosis, model development |
| (Vasa et al., 2024) | 2024 | India | Feasibility | Test Dataset:  Facial Drooping  Detection:960  Speech Difficulty  Evaluation: 300  Arm Weakness detection: 50 | Healthy individuals + Stroke Patients | Indirect | Deep Learning Mobile App | - FAST criteria used for stroke detection through image and speech analysis - Easily accessible even those who might not have immediate access to clinical care | App-based, no clinical visits needed or specialized equipment | Accuracy: 83.84% (facial), 96.33% (speech) | Need validation in real-world clinical environments. |
| (Mat Said et al., 2021) | 2021 | Malaysia | Cluster RCT | Two groups, 66 in each group | One with the app, another with standard management | Indirect | Mobile Application | - The Stroke RiskometerTM app: Stroke risk measurement - personalized warnings, lifestyle correction messages - Easily scalable compared to the traditional public health intervention | Self-management tool | The app has shown modest yet significant improvements in self-efficacy, health behaviors, status, and quality of life at low cost. | Effectiveness studied on a small, culturally specific sample |
| (Shah et al., 2024) | 2024 | India | Feasibility (AI/ML) | 2 stroke prediction datasets: 5110 and 4798 samples | Individuals with health-related factors | Indirect | ML-based Mobile Application | - Clinical Decision Support System (CDSS) App - used Age, BMI, Gender, Heart Disease, and Smoking Status for stroke prediction - Portability: High - Easy access to both Patient side and Doctor side | Patients side and Doctors side. Each side plays a different role and needs to learn the app usage in two different angles. | Random Forest accuracy rate of approximately 89%. | Future model refinement and scaling |
| (Cai et al., 2024) | 2024 | USA | Feasibility (Mobile AI triage) | 269 participants | Mild-moderate stroke patients | Indirect | Multi-Modal Mobile AI tool | - Audio-visual multimodal AI for stroke detection - Lightweight and userfriendly mobile application - High adaptability and easy adaptation to other health conditions | No need | achieves 80.85% accuracy, 60.00% specificity, and 90.63% sensitivity. | - Generalizing framework to different stroke severity. |
| (Chen & Sawan, 2021) | 2021 | China | Narrative Review | N/A | Wearable tech users | Indirect | Wearable Technology | - Real-time monitoring - wristbands are a potentially low-cost and low burden technology | Minimal Instruction for basic wearables; moderate-high for EEG-fNIRS based wearables | Consumer wearables offer continuous monitoring but lower precision  Multimodal EEG-fNIRS high predictive performance  ML and multimodal data fusion enhances reliability | Standardization challenges  Need Clinical Validation |
| (Zeng et al., 2023) | 2023 | China | Feasibility (Wearable Device) | 24 healthy volunteers | Potential  stroke individuals and stroke patients | Indirect | Wearable ECS-based cerebral blood flow monitoring device | - Deep Brain Sensing Real-time brain blood flow and oxygen monitoring - Truly Wearable, Lightweight (60x60 mm), battery powered - Non-invasive - Not bulky - Powered by lithium batteries and can conduct real-time and continuous monitoring | Minimal; user-friendly interface, continuous monitoring in mobile terminal or PC  through wireless communication | High; detected real-time CBF changes in simulations and clinical trials (p < 0.05) | High Potential in stroke warning systems, continuous monitoring, and personalized cerebral health |
| (Sharma et al., 2024) | 2024 | India | Feasibility (ML model) | 3926 training + 982 testing patient records | Stroke Patients | Indirect | ML-based Stroke Prediction Model | Used demographic and health characteristics for early stroke detection  High deployability: software-based, cloud or local systems  Easy integration with any kind of device | Integrable with digital machines | Very high; 94.6% accuracy | Need explainable AI for clinical trust. Proposed multi-modal fusion (genetics, imaging, wearables) for personalized stroke risk prediction. |
| (Yusro et al., 2025) | 2025 | Indonesia | Mixed Methods Design and Evaluation | Not mentioned, Two distinct user groups | younger adults and  elderly users | Indirect | Mobile Health Application (mHealth) | - A user-friendly interface for people with varying levels of literacy and technological - Knowledge Real-time stroke risk prediction based on individual health data entered by the user - Compatibility with Android devices to allow widespread accessibility, as Android devices dominate the smartphone market in many regions | App with user guides and training materials to assist with installation, health data input, and interpreting stroke risk predictions | Younger users reported high satisfaction (88.05%) | Integrating My-Stroke Guard with local healthcare systems  and expanding its device compatibility |
| (Olawade et al., 2025) | 2025 | Global | Narrative Review | Not reported the number of papers | Stroke & wearable tech studies | Indirect | AI-driven wearable devices | AI enables continuous monitoring and personalized stroke care from biometric data.  Subsidized wearables and telemedicine improve access in underserved areas. | Reduces in-person visits, improving stroke prevention access in remote areas. | Continuous monitoring, personalized care, improve stroke assessments | Lacks quantitative methods like meta-analysis, limiting statistical evaluation |

Direct - Pediatric SCD specific

Indirect - Technology-enabling / Adaptable

**Note:** Studies labeled as Direct evaluated stroke screening approaches in pediatric sickle cell disease populations. Studies labeled as Indirect were included as technology-enabling evidence to inform scalable, low-resource screening solutions potentially adaptable to pediatric SCD care and were not interpreted as direct clinical evidence.

**Preprints:** (Nri-Ezedi et al., 2024) This study is a preprint and has not yet undergone peer review.
